# The Structural and Dynamical Effects of Monolignol Coniferyl Alcohol on Biomembranes

**DOI:** 10.1021/acsomega.6c08435

**Published:** 2026-10-28

**Authors:** Shuo Qian, Honghai Zhang, Piotr Zolnierczuk, Eugene Mamontov

**Affiliations:** 6146 Oak Ridge National Laboratory, Oak Ridge 37830, Tennessee, United States

## Abstract

Monolignols are lignin precursors that must cross the plasma membrane before polymerization in the cell wall, but their interaction with lipid membranes remains poorly defined experimentally. In this work, we used Neutron Membrane Diffraction (NMD), Small-Angle Neutron Scattering (SANS), Neutron Spin Echo (NSE), and Quasi-Elastic Neutron Scattering (QENS) to examine the structural and dynamical effects of coniferyl alcohol (CA) in a DOPC/DOPE model membrane. Partially deuterated coniferyl alcohol (CA-D5) provided enhanced neutron contrast for probing changes in the *trans*-bilayer structure and possible distribution within the bilayer. NMD did not resolve CA-D5 at a specific bilayer depth over the nominal concentration range studied. Both NMD measurements of substrate-supported multilamellar bilayers and SANS measurements of unilamellar vesicles in solution revealed a small but clearly resolved bilayer thinning of approximately 1–2 Å, while the overall membrane architecture remained intact. Dynamically, CA-D5 softens the membrane at the collective length scale, as shown by a reduced effective bending modulus from NSE. At the molecular scale, QENS showed no systematic change in lipid lateral diffusivity, although the characteristic microscopic displacement length increased at higher CA-D5 concentrations. These cross-scale measurements show that CA produces measurable but limited changes in membrane structure and dynamics without major membrane reorganization. This membrane-compatible response is consistent with various proposed monolignol transport pathways, including passive permeation and vesicle-associated transport.

## Graphical abstract

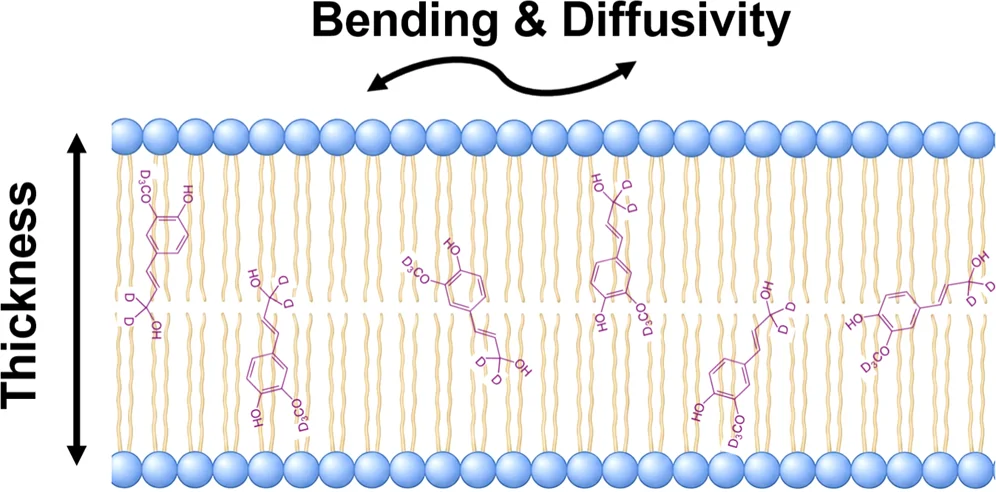

## Introduction

Lignin is the second most abundant natural biopolymer, accounting for 25% of wood biomass after cellulose, and is an increasingly important source of renewable chemicals for energy and industry applications. In plants, it provides rigidity and protection to the secondary walls of xylem cells, enabling efficient water transport and structural support.^1^ Its precursors, monolignols, are synthesized in the cytoplasm via the phenylpropanoid pathway. They must cross the plasma membrane to the apoplast to be polymerized by laccases and peroxidases to form lignin. The mechanism of this transport remains under investigation, with proposed pathways including active transport by proteins such as ATP-binding cassette (ABC) transporters^2^ and passive diffusion through cell membranes driven by concentration gradient created during the polymerization process in the cell wall.^3–5^ Another recent study also suggested that extracellular vesicles participate in transporting monolignols along with the enzymes required for polymerization in Norway spruce, potentially enabling radical coupling during transit.^6^

In addition, monolignols have a strong propensity to interact with and embed within cell membranes, making it essential to understand their effects on the membrane structure and function. Elevated monolignol concentrations could disrupt the membrane integrity and increase its overall permeability. This might disturb the cellular homeostasis and interfere with physiological processes. Understanding their effects on membranes is crucial, not only for elucidating the mechanism of monolignol transport but also for advancing strategies to use bacteria to depolymerize lignin into value-added chemicals such as biofuels, chemical feedstocks, antioxidants, and antimicrobials.^7^

Monolignols are capable of diffusing across the hydrophobic core of lipid bilayers that act as a barrier between the intracellular and extracellular environments.^3,5^ As they diffuse, the resulting structural and dynamical responses in membranes remain poorly understood experimentally. In this work, we investigate how the presence of monolignols influences membrane structure, mechanical properties, and lipid dynamics. Specifically, we examine whether monolignols modify bilayer thickness or internal organization, and how such changes affect membrane bending rigidity and lateral lipid diffusivity at the molecular scale. Understanding their effect on membranes, while it will not provide direct evidence for their transport pathways, answers a central physical question pertinent to how monolignols overcome the cellular compartmental barrier.

We employed a series of neutron-scattering techniques to address these questions at the molecular level. Compared to other structural probes such as X-ray scattering and electron and light microscopy, neutron scattering is particularly sensitive to light elements and their isotopes, most notably demonstrated by the large difference in scattering cross sections of hydrogen isotopes (protium and deuterium). This enables contrast variation through isotopic substitution to selectively highlight specific components.^8,9^ In this study, we used partially deuterated coniferyl alcohol (CA) to probe its distribution within lipid membranes and its effect on the membrane structure at varying nominal concentrations. Neutron membrane diffraction (NMD) was first applied to resolve the *trans*-bilayer structure with and without CA. Small-angle neutron scattering (SANS), a bulk technique capable of covering a few to hundreds of nm in length scale, was then used to characterize the bilayer structure of unilamellar vesicles in solution.

To probe membrane dynamics on a few to hundreds of nanometers, neutron spin echo (NSE) measurements were performed to quantify collective membrane fluctuations up to submicrosecond time scale and extract bending rigidity. This allows us to assess how CA influences membrane mechanical properties as a function of the concentration. At the scale of a few angstroms and nanometers, quasielastic neutron scattering (QENS) was used to examine lipid lateral diffusion from picoseconds to nanoseconds. By correlating structural and dynamical measurements, we determine how CA affects lipid mobility and membrane properties, which are important for understanding the membrane function and monolignol transport behavior.

Our results show that CA causes small thinning of membrane bilayers while keeping the overall structure intact. There is no localized accumulation of CA found inside the bilayers. At the same time, NSE reveals a slight softening of the membrane once CA is present, while QENS indicates that local lipid diffusivity shows no sign of systematic change, with only subtle extensions in transport length scales at higher concentrations. Together, these results suggest that the membrane environment remains structurally and dynamically compatible with, but not direct evidence for, proposed monolignol transport pathways such as passive permeation and vesicle-associated transport.^3–6^ The work also demonstrates how neutron scattering can provide a molecular-level understanding of lignin-related compounds’ interaction with biomembranes across multiple length and time scales, informing strategies to manipulate lignin biosynthesis and breakdown for biomass utilization and bioenergy applications.

## Materials and Methods

### Materials

1,2-Dioleoyl-*sn*-glycero-3-phosphocholine (DOPC) and 1,2-dioleoyl-*sn*-glycero-3-phosphoethanolamine (DOPE) lipids were purchased from Avanti Polar Lipids (Birmingham, Alabama) and used as received (Figure 1a,b). Deuterium-labeled coniferyl alcohol, CA-D5, (Figure 1c) was synthesized in-house following established chemical deuteration procedures, and its structure and labeling were confirmed prior to use (please see Supporting Information for details).

**Figure 1:**
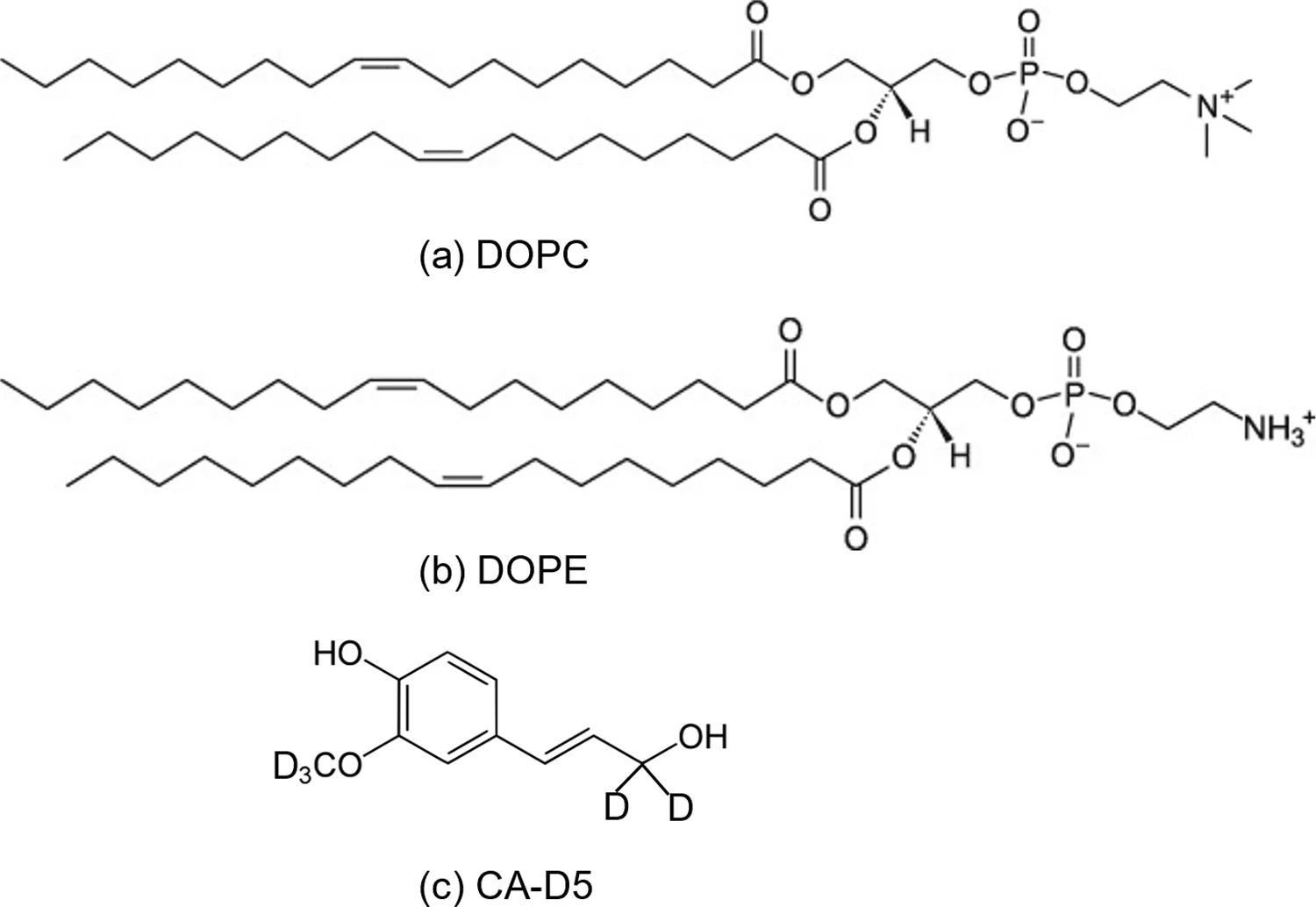
Chemical structures of (a) DOPC, (b) DOPE, and (c) CA-D5.

### Membrane Diffraction Experiment and Data Analysis

For the neutron membrane diffraction experiment, DOPC/DOPE = 1/1 (molar ratio) was mixed with CA-D5 of molar ratios 0, 5, 10, 15, and 25 (only for QENS) mol % in a 1:1 (v/v) solution of trifluoroethanol and chloroform. Following the established method, the mixture containing about 5 mg of lipids was deposited onto each clean quartz substrates (19 mm × 38 mm).^10–12^ After the organic solvent evaporated in air, the lipid film on the substrate was visually uniform. The samples were placed under vacuum to remove any residual organic solvent and then rehydrated in a sealed chamber containing saturated D_2_O vapor at 30 °C for at least 24 h to improve their multilamellar alignment. Two stacking substrates for each sample were used in the measurement to strengthen signals.

Neutron membrane diffraction measurements were carried out at the EQ-SANS instrument at the Spallation Neutron Source (SNS) of Oak Ridge National Laboratory (ORNL). The instrument was operated with a wavelength band of 2.5–6.8 Å defined by the chopper system and a sample-to-detector distance of 1.3 m. Samples were sealed in a humidity-controlled cell and positioned at grazing incidence relative to the incoming beam.^10^ Temperature was maintained at 25 °C throughout the experiment. The sample relative humidity (RH) was regulated with a humidity generator (Model RH-200, L&C Science, Hialeah, FL, USA) with an 8% (v/v) D_2_O reservoir. At 8% D_2_O, hydration water contributes approximately zero net coherent scattering amplitude. Consequently, at this condition, diffractions measured at different lamellar repeat spacings sample a common continuous transform of the bilayer structure, enabling phase assignment by the swelling method accurately.^13^

After allowing sufficient time for equilibration under these conditions, diffraction data were acquired by performing a rocking ω-scan. In this measurement, the sample was incrementally rotated from 0° to 12° with a step size of 0.1° for the full diffraction pattern.

The data reduction and analysis followed established methods.^10–12,14^ Briefly, the diffraction data were first reduced to two-dimensional reciprocal space maps (*Q*_*r*_, *Q*_*z*_) using the drt-SANS package,^15^ with standard corrections applied for detector and instrument effects, including proton charge normalization, pixel sensitivity, and dark current subtraction. Diffraction peaks were then integrated over the 2D maps to obtain the intensity.

To extract the structure factor amplitudes |*F*|, the integrated intensities (*I*) were corrected for the Lorentz factor (*C*_L_), the sample absorption (*C*_abs_), and the sample geometry (*C*_geo_) (see their detailed definitions and equations in the Supporting Information):

$$\left|F\right|=\sqrt{I/I_{0}C_{L}C_{abs}C_{geo}}$$

A total of five diffraction orders were observed (Figure S2). The lamellar repeat spacing, *D*, was determined from the peak positions. Because the lipid bilayer is centrosymmetric, the structure factor phases are restricted to ±1. The phases were determined using the swelling method^13,14,16^ (Figure S3, see Supporting Information for details.).

The neutron scattering length density (SLD) profile along the bilayer normal, ρ­(*x*), was constructed from *F*(*h*) by

$$\rho \left(x\right)=\sum_{h}F\left(h\right)\;\cos\left(2\pi hx/D\right)$$

where *h* is the Miller index (*h* = 1 to 5).

Because no absolute reference was available for scaling the diffraction amplitudes,^10,12,17^ the reconstructed SLD profiles are presented on a relative rather than absolute scale and are therefore interpreted qualitatively. To account for small differences in sample amount within the beam, the structure factor amplitudes from each sample were normalized before reconstruction by

$${\left[\sum_{h}F^{2}\left(h\right)\right]}^{1/2}/D$$

This normalization places the profiles on a common relative scale for the comparison of their shapes and internal structural features.

### SANS Experiment and Data Analysis

Large unilamellar vesicles (LUVs) were used for SANS, NSE, and QENS experiments. After complete removal of the organic solvent from the DOPC/DOPE/CA-D5 cosolution under vacuum, the resulting mixtures were dispersed in D_2_O to a final concentration of 15 mg/mL. After vigorous vortexing, the suspensions were subjected to a minimum of three freeze–thaw cycles by alternating between 30 °C and −80 °C. LUVs were subsequently prepared using an Avanti mini-extruder (Alabaster, Alabama) fitted with 0.1 μm pore-size membranes. The samples were passed through the membrane 17 times to achieve unilamellarity and uniform vesicle size. Prepared LUV solutions were stored at 4 °C and used within 1 week if not measured immediately. The lipid solutions were loaded into quartz circular cells with a 1 mm beam path (Hellma, Germany) for SANS measurements at 25 °C, well above the lipid phase transition temperatures.

The SANS measurements were performed using the EQSANS instrument.^18^ Two instrument configurations were used: sample-to-detector distance of 4 m, wavelength band of 10–13.6 Å and sample-to-detector distance of 2.5 m, wavelength band of 2.5–6.5 Å both at 60 Hz operation mode. Using drt-SANS, the data were then corrected for transmission and background scattering from solvent and quartz cells and azimuthally averaged into 1D scattering curves (*I* vs *Q*), where *I* is the scattering intensity arising from the contrast between the LUVs and D_2_O, and *Q* is the scattering vector defined as *Q* = 4π sin θ/λ, where θ is half of the scattering angle and λ is the neutron wavelength. The instrument resolution was provided with the data and was used in the SANS data fitting to account for the instrumental smearing effect.

The data fitting was performed in SASView software (version 6.0.3, sasview.org). The SANS data were analyzed using a core–multishell model with three shells appropriate for unilamellar vesicles for the *Q* range 0.01 to 0.35 Å^–1^. The SLDs of both the solvent and the vesicle core were fixed to that of D_2_O (6.34 × 10^–6^ Å^–2^), while the first and third shell SLD was set to 2.0 × 10^–6^ Å^–2^, an average value for DOPE/DOPC inner and outer headgroups. The second shell as the hydrocarbon chain was set to −0.4 × 10^–6^ Å^–2^, representative of an average protiated lipid chain. The vesicle radius was fixed at 500 Å with an overall size polydispersity of 0.25.^19^ The parameter uncertainties reported from SASView software were estimated from the square roots of the diagonal elements of the covariance matrix produced by the Levenberg–Marquardt optimizer, therefore they are fitting uncertainties rather than experimental uncertainties.

### NSE Experiment and Data Analysis

NSE experiments were carried out at the NSE instrument (BL-15) at SNS.^20^ The samples were kept at 25 °C with a precision of ±0.5 °C during measurement using quartz cells of 3 cm × 3 cm × 4 mm. Two neutron wavelength bands (5–8 Å and 8–11 Å) were selected to access Fourier times from approximately 0.07 to 100 ns over a *Q* range of ∼0.055–0.1 Å^–1^. Background scattering was subtracted using D_2_O measurements collected in identical sample cells. Data reduction was performed using the DrSPINE program.^21^

Following the model developed by Zilman and Granek,^22^ fluctuations of a freestanding single-membrane were modeled to fit the intermediate scattering function

$$\frac{S\left(Q,t\right)}{S\left(Q,0\right)}=e^{-{\left(\Gamma \left(Q\right)t\right)}^{2/3}}$$

where *t* is the Fourier time. From these fits, the *Q*-dependent relaxation rate Γ­(*Q*) was extracted. The effective bending modulus κ̃ was then determined using the relation proposed by Watson and Brown:^23^

$$\Gamma \left(Q\right)=0.025\gamma \sqrt{\frac{k_{B}T}{\mathrm{\kappa ̃}}}\frac{k_{B}T}{\eta_{D_{2}O}}Q^{3}$$

where η_D_2_O_ is the viscosity of D_2_O at 25 °C. The prefactor γ is taken to be unity under the condition κ̃ ≫ *k*_B_ *T*, which is typically satisfied for lipid bilayers near room temperature.^22,24,25^

### QENS Experiment and Data Analysis

The solution samples were loaded into cylindrical annular 0.5 mm thick aluminum sample holders (an outer diameter of 29 mm, an inner diameter of 28 mm, and a height of 54 mm). The geometry of the sample holders was chosen to ensure greater than 90% neutron transmission through the samples to reduce multiple scattering. The QENS experiments were carried out at the SNS backscattering spectrometer, BASIS.^26^ A standard spectrometer configuration was used providing the analyzable energy transfer, *E*, range of ±100 μeV. The energy resolution at the elastic line was 3.0 μeV (fwhm, full width at half-maximum). The accessible momentum transfer range was 0.3 Å^–1^ < *Q* < 1.9 Å^–1^. All the samples as well as the D_2_O buffer sample were measured at 25 °C. Standard data reduction was carried out using the Mantid software package.^27^ After subtraction of the D_2_O buffer background data from the measured spectra, the resulting spectra were fitted (at each *Q* individually) using the DAVE software package^28^ with the following model:

I(Q,E)=S(Q,E)⊗R(Q,E)+B(Q,E)

That is, the model dynamic structure factor, *S*(*Q*, *E*), which represents the dynamics of the lipids, was numerically convolved with the sample-specific resolution function, R­(*Q*, *E*), measured at 10 K. The background term, B­(*Q*, *E*), was linear in energy transfer: B­(*Q*, *E*) = C_1_(*Q*) + C_2_(*Q*, *E*). Following the typical approach utilized in QENS measurements of lipid vesicles, we used the model dynamic structure factor as a sum of two Lorentz functions:

$$S\left(Q,E\right)=x\left(Q\right)\frac{1}{\pi }\frac{\Gamma_{n}\left(Q\right)}{{\Gamma_{n}\left(Q\right)}^{2}+E^{2}}+\left[1-x\left(Q\right)\right]\frac{1}{\pi }\frac{\Gamma_{b}\left(Q\right)}{{\Gamma_{b}\left(Q\right)}^{2}+E^{2}}$$

The broad Lorentz function component with half width at half-maximum Γ_b_ accounts for the fast localized lipid dynamics. The narrow Lorentz function component with half width at half-maximum Γ_n_ is associated with the lateral diffusion of lipid within the bilayer.

## Results and Discussion

### Coniferyl Alcohol Incorporation Induces Small Bilayer Thinning and Modifies the *trans*-Bilayer SLD Profile

The model membrane in our study is composed of phosphatidylcholine (PC) and phosphatidylethanolamine (PE), two major phospholipids found in plant cell membranes.^29^ Specifically, the lipid composition DOPC/DOPE = 1/1 (molar ratio) was used for all experiments. CA-D5 was added to the lipid in molar ratios of 5%, 10%, and 15%. In the NMD experiment, the samples were a substrate-supported multilamellar stack of lipid bilayers. At slightly lower than full hydration (RH 98% and 95%), the well-aligned samples showed only one series of excellent lamellar diffraction peaks up to five orders (Figure S2) without any sign of additional ordered phases. Therefore, it is unlikely that added CA-D5 formed a separate ordered phase outside the bilayer. The data provided high-quality structural information perpendicular to the plane of the lipid bilayer, hence the *trans*-bilayer structure. The measurements were carried out in 8% D_2_O, where the SLD of water is effectively zero due to cancellation between hydrogen, deuterium, and oxygen contributions.^12^ This contrast minimizes the contribution from hydration water, enabling an accurate phase determination by the swelling method and a more direct interpretation of the lipid bilayer structure.

From the peak positions, the lamellar repeat spacing (*D* spacing) was determined. As summarized in Table 1, the *D* spacing remains essentially unchanged upon CA-D5 addition at RH 98%, with 56.9 ± 1.5 Å for the lipid only sample and 56.9–57.0 Å across all CA-D5 concentrations. However, structural changes are evident in the relative diffraction peak intensities from the diffraction patterns, particularly the weakening of the fourth-order peak upon CA-D5 incorporation (Figure S2), suggesting that CA-D5 indeed incorporates into the bilayer and modifies the internal SLD.

**Table 1:** Structural Parameters from SLD Reconstructed from NMD at RH 98%

sample	*D* spacing (Å)	peak-to-peak distance (Å)
lipid only	56.9 ± 1.5	36.9 ± 1.0
+ 5% CA-D5	57.0 ± 1.4	35.3 ± 0.9
+10% CA-D5	56.9 ± 1.2	35.2 ± 0.7
+15% CA-D5	56.9 ± 1.0	35.4 ± 0.6

The SLD profiles for all the samples at RH 98% were reconstructed from the diffraction amplitudes and phases (Table 2), with phases determined using the swelling method (see Supporting Information and Figure S3 for details). The reconstructed profiles (Figure 2) show the lipid bilayer structure changes upon CA-D5 addition. In the SLD profiles, the two major peaks correspond to the interfacial headgroup/glycerol regions of the bilayer, where the average SLD is relatively high. The central trough represents the hydrocarbon core, which has a lower SLD due to the protiated lipid chains. The peak-to-peak distance, a measure of bilayer thickness, decreases slightly from 36.9 Å in the lipid-only sample to approximately 35.2–35.4 Å with CA-D5. This corresponds to a modest thinning of the bilayer, indicative of a small increase in chain disorder. Notably, this thinning does not show a clear dependence on the nominal CA-D5 loading.

**Table 2:** Diffraction Amplitudes after Normalization (Arbitrary Units) and Phases

sample	*F*(1)	*F*(2)	*F*(3)	*F*(4)	*F*(5)
lipid only	4.80 ± 0.12	3.57 ± 0.12	2.16 ± 0.15	1.38 ± 0.12	1.44 ± 0.14
+5% CA-D5	4.62 ± 0.14	3.86 ± 0.13	2.38 ± 0.14	0.12 ± 0.01	1.62 ± 0.12
+10% CA-D5	4.57 ± 0.10	4.08 ± 0.10	2.43 ± 0.14	0.12 ± 0.01	1.59 ± 0.21
+15% CA-D5	4.69 ± 0.12	3.92 ± 0.10	2.24 ± 0.15	0.10 ± 0.01	1.45 ± 0.19
phases	–1	–1	+1	–1	–1

**Figure 2:**
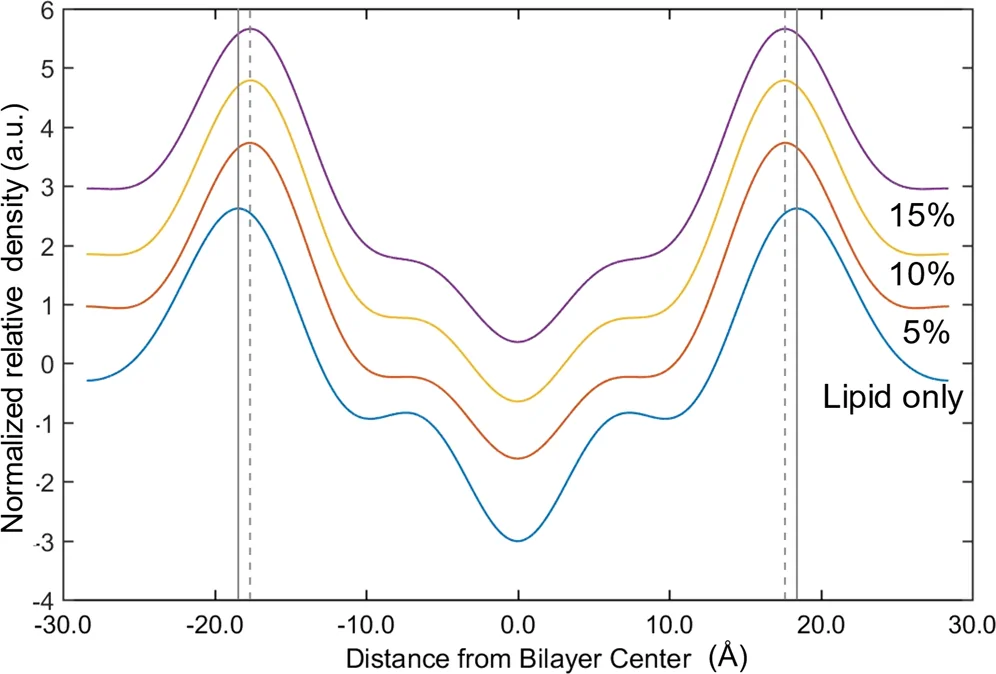
Reconstructed SLD profiles along the normal direction of the lipid bilayer at RH 98%. They are offset for clarity (the overlaid profiles are shown in Figure S4). The vertical gray lines show the peak positions of the lipid-only sample, and the vertical dash lines show those of the CA-D5-added samples approximately.

From the SLD profiles, more localized structural changes are observed in the hydrocarbon region. The characteristic feature associated with the double-bond kink in DOPC and DOPE chain region, located ∼8 Å from the bilayer center,^30^ is reduced in amplitude upon CA-D5 addition. This suggests the perturbation of chain packing near the unsaturated region.

Partially deuterated CA-D5 was used to provide additional neutron contrast for probing its spatial distribution within the bilayer. However, as mentioned in the Materials and Methods, the lack of an absolute scale in SLD prevents a quantitative comparison of SLD differences among profiles. Within these limitations, the reconstructed SLD profiles show no distinct localized feature or concentration-dependent “bump” that would indicate preferential accumulation of CA-D5 at a specific bilayer depth as nominal CA-D5 concentration increased. Overall, the NMD results show that CA incorporation leads to detectable modifications in lipid chain packing along with a small thinning effect.

SANS measurements were performed on unilamellar vesicles composed of DOPC/DOPE = 1:1 (molar ratio) in 100% D_2_O with the same nominal concentrations of CA-D5. Under these conditions, the strong contrast between the protiatedlipids and the D_2_O solvent enhances sensitivity to detect structural variations within the bilayer.

The scattering profiles (Figure 3) exhibit features characteristic of dilute LUV solutions. In the low-*Q* region, the intensity follows an approximate *Q* ^–2^ dependence, consistent with scattering from sheet-like bilayer structures in unilamellar vesicles.^31^ At higher *Q*, the presence of a pronounced minimum, followed by a broad peak, reflects the form factor of a well-defined bilayer.

**Figure 3:**
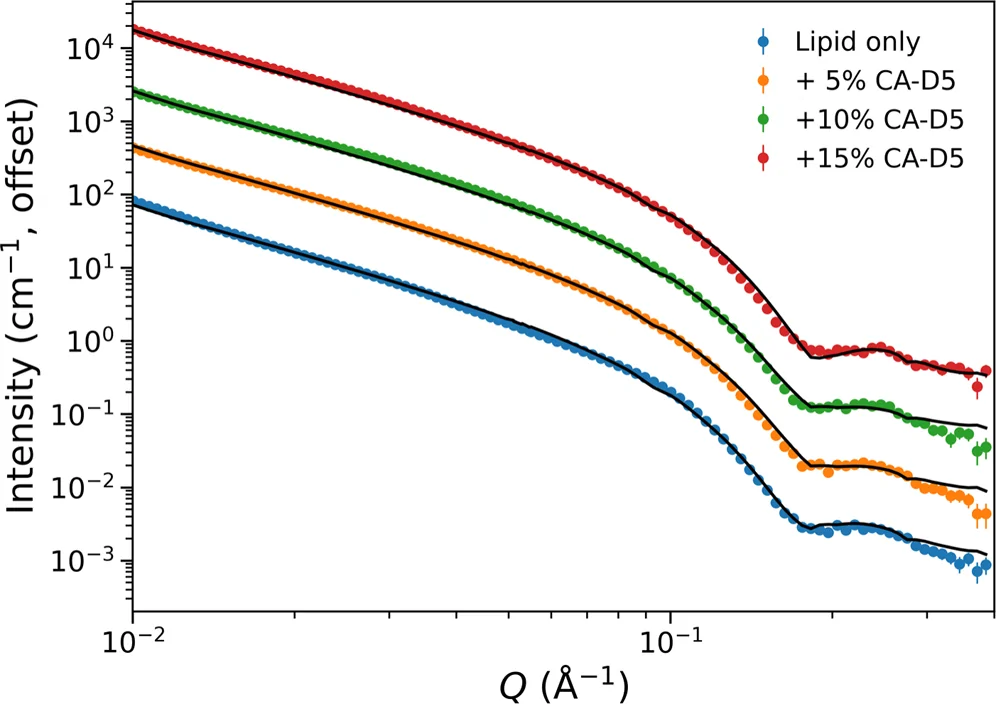
SANS data from LUV, with offset for clarity. See Figure S5 for overlaid data.

Estimates of the characteristic bilayer form factor length scale can be obtained directly from these features. For example, the position of the peak at *Q* ≈ 0.22 ± 0.0055 Å^–1^ for the lipid-only sample yields an estimated length scale 2π/*Q* = 28.6 ± 0.7 Å. Adding CA-D5 to the bilayer resulted in a small yet noticeable reduction to 27.5 ± 0.7 Å for 5% and 10% CA-D5 and 25.5 ± 0.6 Å for 15% CA-D5.

Along with that, a systematic and modest decrease in scattering intensity is observed across the low-to-intermediate *Q* range (0.01 Å^–1^ < *Q* < 0.1 Å^–1^) with increasing CA-D5 concentration (see the overlaid plot in Figure S5), with the lipid-only sample exhibiting the highest intensity and the 15% CA-D5 sample the lowest. This trend is consistent with contrast reduction arising from the incorporation of deuterated CA, which increases the average SLD of the membrane and reduces the contrast relative to the D_2_O solvent. Closer inspection of the high-*Q* region shows that the minimum and peak features become slightly less well-defined at the 15% CA-D5 sample. This may reflect more reduced contrast associated with higher nominal CA loading.

The SANS profiles were fitted using a core–multishell sphere model containing separate inner-headgroup, hydrocarbon-chain, and outer-headgroup shells, with the fitted curves shown as solid black lines in Figure 3. The resulting structural parameters are summarized in Table 3. The fitted inner- and outer-headgroup regions are asymmetric, with effective thicknesses of approximately 9–10 Å and 5 Å, respectively. These values vary relatively little with the nominal CA-D5 concentrations. Such asymmetry may arise from different intrinsic curvatures of the lipids, with DOPE having a considerably more negative spontaneous curvature than DOPC. However, because the shell SLDs were fixed and the thickness parameters were correlated within the model, the fitted asymmetry should not be interpreted directly as evidence for preferential DOPC/DOPE distribution between the two leaflets.

**Table 3:** Structural Parameters from SANS Data Fitting

sample	inner-headgroup thickness (Å)	chain thickness (Å)	outer-headgroup thickness (Å)	total thickness (Å)
lipid only	9.40 ± 0.04	26.05 ± 0.05	5.00 ± 0.04	40.45 ± 0.08
+5% CA-D5	9.66 ± 0.04	24.36 ± 0.05	5.10 ± 0.04	39.12 ± 0.07
+10% CA-D5	9.70 ± 0.05	24.40 ± 0.05	5.00 ± 0.06	39.10 ± 0.09
+15% CA-D5	9.10 ± 0.04	24.20 ± 0.05	5.05 ± 0.04	38.35 ± 0.08

In contrast to the relatively stable headgroup regions, the hydrocarbon-chain thickness shows a clear reduction upon CA-D5 addition. The chain thickness decreases from 26.05 ± 0.05 Å for the lipid-only sample to 24.36 ± 0.05 Å at 5% CA-D5 and remains near 24.2–24.4 Å at higher concentrations. Consequently, the total fitted bilayer thickness decreases from 40.45 ± 0.08 Å to 39.12 ± 0.07 Å, 39.10 ± 0.09 Å, and 38.35 ± 0.08 Å at nominal CA-D5 loadings of 5%, 10%, and 15%, respectively. This corresponds to an overall thinning of approximately 1–2 Å or 3–5% of the original bilayer thickness. The change occurs largely upon the initial addition of CA-D5, with similar thicknesses at 5% and 10%, followed by additional thinning at 15%.

Although the numerical uncertainties from fitting are small, and the observed differences are well beyond the fitting uncertainties, the absolute shell dimensions should be interpreted cautiously. The shell SLDs were constrained during fitting to reduce the number of free parameters, and correlations between SLD and shell thickness limit the uniqueness of the fitted dimensions. Therefore, the thickness values are most useful for comparing relative structural changes under identical constraints rather than as absolute dimensions. Nevertheless, the systematic displacement of the scattering profile peaks together with the model fits provide consistent evidence for bilayer thinning upon CA-D5 incorporation.

Importantly, this thinning is consistent with the NMD results. NMD shows a reduction of approximately 1.5–1.7 Å in the Peak-to-Peak distance after CA-D5 addition, while SANS yields a 1.3–2.1 Å decrease in the total thickness. The absolute thicknesses from the two techniques are not expected to be identical because NMD measures different SLD features in partially hydrated oriented multilayers, whereas SANS results are based on a model of fully hydrated vesicles in solution. Their agreement in both the direction and magnitude of the change therefore provides strong evidence that CA-D5 induces a small, relative to the ∼40 Å total bilayer thickness, but detectable thinning of the membrane, while there is no larger structural disruption observed for other membrane-interacting molecules such as drug molecules, peptides, or nanoparticles.^9,11,25,32^ The combined NMD and SANS results provide a structural baseline for the dynamical studies. We therefore turn to NSE and QENS to probe how CA influences membrane fluctuations and lipid mobility.

### Coniferyl Alcohol Softens Bilayer while Largely Preserving Lipid Diffusivity

NSE probes collective out-of-plane membrane fluctuations on the nanosecond time scale and provides access to an NSE-derived effective bending modulus κ̃, while QENS measures lipid motions at the molecular scale, including lateral diffusion and localized relaxation. The NSE experiment used the lipid-only, 10%, and 15% CA-D5-added samples. The NSE results show that CA-D5 softens the lipid membrane (Figure 4; Table 4). The effective bending modulus of the lipid-only membrane was 297 ± 12 *k*_B_ *T*. With 10% CA-D5 added, κ̃ decreased to 209 ± 8 *k*_B_ *T*, corresponding to ∼70% of the lipid-only value. Increasing the CA-D5 concentration to 15% further reduced κ̃ only slightly to 192 ± 11 *k*_B_ *T* or ∼65% of the lipid-only membrane (Figure 4). This behavior is similar to the trends in the structural results from NMD and SANS, where CA induces small bilayer thinning and internal structural changes once incorporated. Most of these changes appear upon the initial incorporation of CA-D5; however, we caution that the limited number of data points does not allow us to establish a definitive saturation effect.

**Figure 4:**
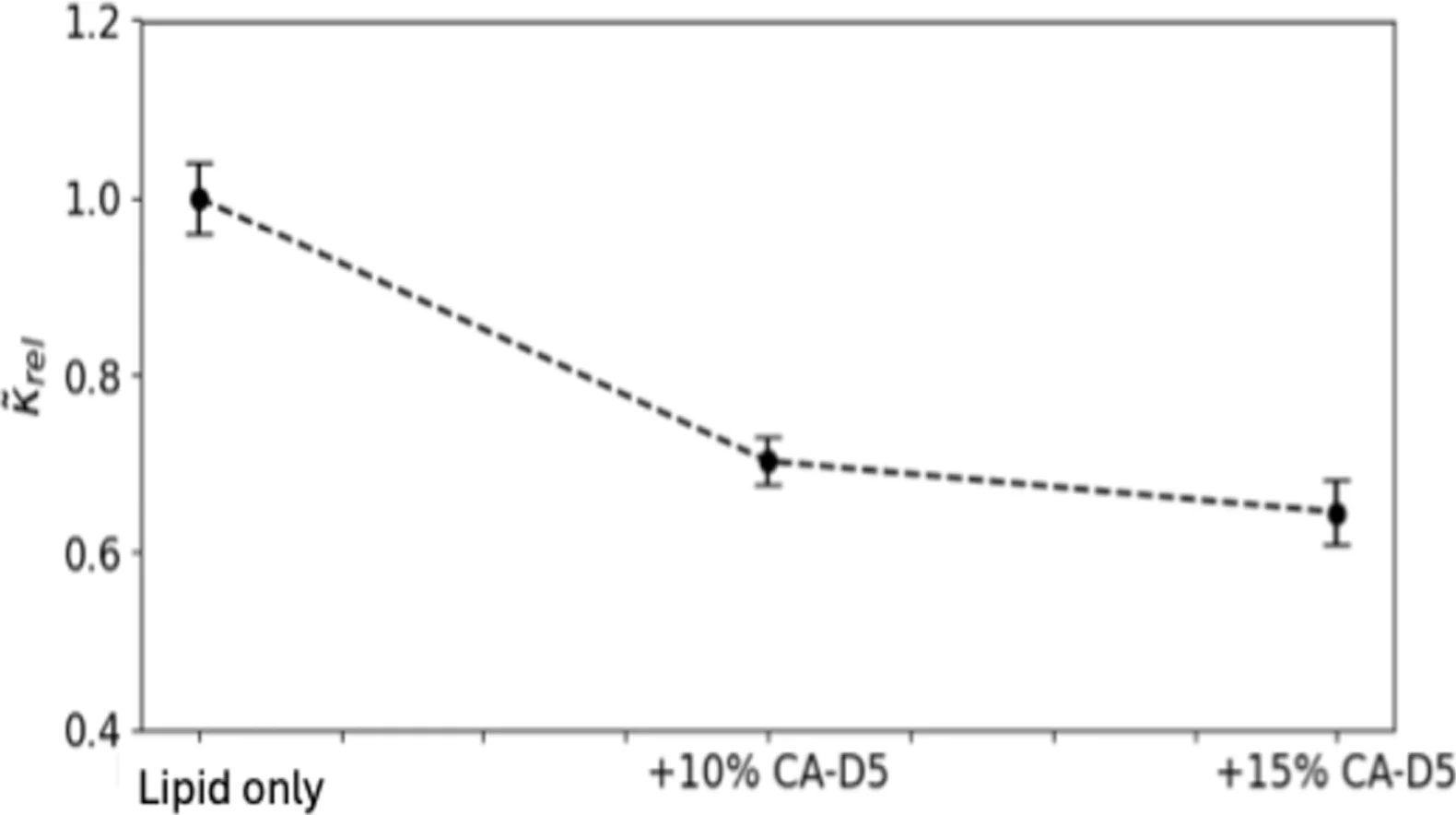
Membrane effective bending modulus relative to the lipid only sample as ${\mathrm{\kappa ̃}}_{rel}={\mathrm{\kappa ̃}}_{sample}/{\mathrm{\kappa ̃}}_{lipidonly}$ .

**Table 4:** Membrane Effective Bending Modulus, κ̃, Lipid Lateral Diffusion Coefficient, *d*, and the Jump Length, L

sample	κ̃ (*k*_B_ *T*)	*d* (10^–10^ m^2^/s)	*L* (Å)
lipid only	297 ± 12	0.358 ± 0.010	1.36 ± 0.20
+10% CA-D5	209 ± 8	0.422 ± 0.015	1.23 ± 0.33
+15% CA-D5	192 ± 11	0.366 ± 0.050	3.24 ± 0.48
+25% CA-D5	N.A	0.447 ± 0.027	3.44 ± 0.20

Note that the modulus value here should also be viewed as an NSE-derived effective stiffness rather than necessarily the equilibrium Helfrich bending modulus. Recent works show that vesicle size, translational diffusion, membrane tension, membrane viscosity, and finite bilayer thickness can affect the relaxation measured within the NSE time window.^33,34^ Therefore, the relative reduction among samples prepared and analyzed here under the same conditions is the most direct observation, while quantitative comparison of the absolute values with moduli measured by equilibrium mechanical methods requires caution.

QENS was then used to determine whether this mesoscale softening is accompanied by changes in microscopic lipid mobility. Here, the neutron beam time availability allowed us to measure an additional sample with 25% CA-D5 added. The *Q*-dependent line widths, plotted as HWHM versus *Q*, show that the low-*Q* region is similar across the samples, indicating that the apparent lipid lateral diffusivity is not strongly or systematically affected by CA-D5 (Figure 5). However, the high-*Q* data behavior differs from many reported QENS measurements of lipid lateral diffusion, where the data can often be described by a continuous Fickian diffusion model

$$\Gamma \left(Q\right)=\hbar dQ^{2}$$

where *d* is the lateral diffusion coefficient. Instead, all four samples require a jump diffusion description, similar to recent observations for ergosterol in POPC membranes.^12^ The data were therefore fitted using

$$\Gamma \left(Q\right)=\frac{6\hbar d}{L^{2}}\left(1-\frac{\sin\left(QL\right)}{QL}\right)$$

where *L* is the finite jump distance. In the limit of *L* → 0, this expression reduces to the Fickian diffusion model.

**Figure 5:**
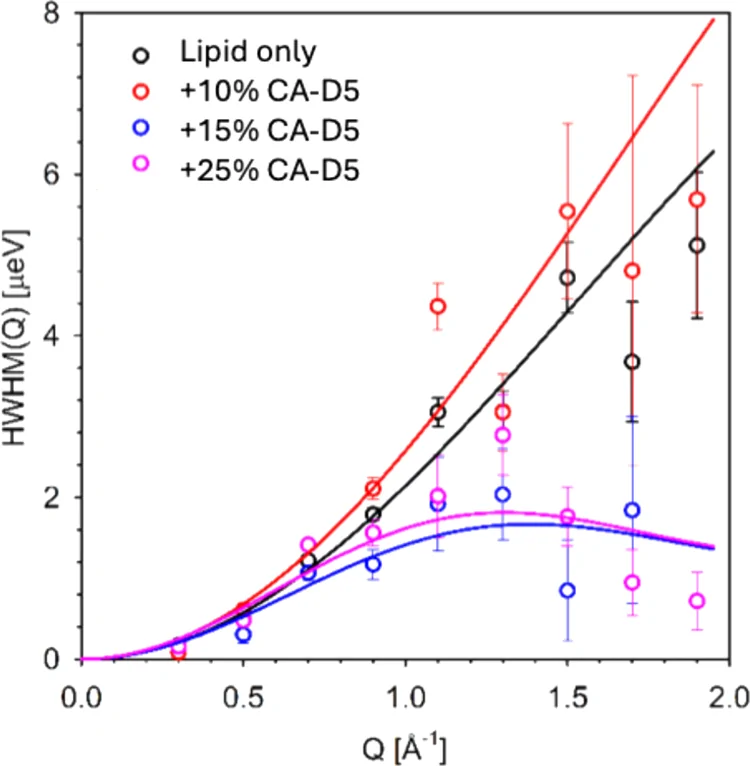
*Q* dependence of the HWHM of the narrow Lorentzian component associated with lipid lateral relaxation. Solid curves are fits to a finite jump diffusion model.

The fitted diffusion coefficients show no clear monotonic dependence on CA-D5 concentration (Table 4). The lipid-only membrane gives *d* = 0.358 ± 0.010 × 10^–10^ m^2^/s, while the 10%, 15%, and 25% CA-D5 samples give between 0.366 and 0.447 × 10^–10^ m^2^/s, without clear trend of changing. Thus, CA does not appear to substantially alter the local lipid fluidity or produce progressive acceleration of lipid lateral diffusion.

In contrast, the fitted jump distance shows a clear change between the 10% and 15% CA-D5 samples. The lipid-only and 10% CA-D5 samples have similar jump distances of 1.36 ± 0.20 Å and 1.23 ± 0.33 Å, respectively. In the 15% and 25% CA-D5 samples, the jump distance increases to 3.24 ± 0.48 and 3.44 ± 0.20 Å, respectively. Note that a lipid jump here does not necessarily involve exchanging positions with a neighboring lipid. It may instead correspond to relocation into a transient void within the lipid matrix, and the associated jump distance therefore need not be commensurate with the nearest-neighbor distance. This difference is also visible directly from the *Q*-dependent line widths: the low-*Q* data, corresponding to the long-range diffusivity, remain comparable, while the high-*Q* region deviates more strongly for the 15% and 25% samples (Figure 5). Again, this is more like concentration-dependent evolution rather than an abrupt change of the diffusion mechanism, because even the lipid-only sample already exhibits a qualitative breakdown of the continuous diffusion model in favor of the jump diffusion model. Therefore, CA does not mainly change the long-range lipid diffusivity, but it modifies the characteristic length scale of lipid motion at higher concentration. Because *d* = *L* ^2^/(6τ) in the jump diffusion model, the increase in *L* at 15% and 25% CA-D5 implies a longer residence time between jumps rather than simply faster lipid motion. This suggests that individual lipids do sense the presence of CA, but the effect is expressed through the microscopic jump process instead of the long-time diffusion coefficient.

Taken together, NSE and QENS reveal a separation between the collective membrane mechanics and molecular lipid mobility. CA reduces the effective bending rigidity of the membrane, indicating that the bilayer becomes more compliant on larger length scales. However, the lipid lateral diffusivity remains largely preserved, showing that CA does not strongly fluidize the membrane on the individual lipid scale. The increase in jump length at 15% and 25% CA points to a gentle reorganization of the local diffusion landscape but not to a loss of lipid order or membrane integrity.

It is likely that CA-D5 causes a modest but evident change in hydrocarbon-chain packing, producing the thinning detected by both NMD and SANS. The thinning can be associated with increased chain disorder that contributes to the reduced membrane stiffness measured by NSE. However, the thickness reduction alone here is unlikely to explain the full ∼30–35% decrease in effective stiffness. Therefore, we caution that bending rigidity depends on both membrane mechanical thickness and area compressibility for simple fluid vesicle bilayers; therefore, thinning can contribute to softening but does not necessarily account for the entire mechanical response.

At higher nominal CA loading, we postulate that the local lipid environment undergoes an additional dynamical crossover. The characteristic local displacement becomes larger, but the corresponding correlation time also becomes longer, so the apparent long-range diffusion coefficient shows no systematic change. A plausible microscopic picture is that CA modifies the distribution of transient voids and local barriers within the membrane. Lipids may remain in a local environment for a longer time and then participate in a larger rearrangement when sufficient transient free volume develops and hence longer jump distances.

### Implications for Monolignol Transport and Membrane Compatibility

Monolignol transport across the plasma membrane has been discussed in terms of several possible mechanisms including passive permeation, transporter-mediated export, and vesicle-associated transport. Our study addresses a central physical question underlying all of these models: whether a monolignol can be accommodated by a lipid membrane without major restructuring of membranes. The neutron scattering results show that CA-D5 produces a clear but relatively small thinning of the lipid bilayer while preserving the overall membrane architecture and lipid lateral diffusivity. At the same time, CA-D5 makes the membrane softer and modifies the microscopic length scale of the lipid motion at higher concentrations. These results indicate that CA alters membrane packing and collective mechanics without causing major structural or dynamical disruption. This points to the fact that the lipid matrix provides a compatible environment for CA incorporation.

This picture is consistent with molecular dynamics simulations showing that neutral lignin-related phenolics, including monolignols, can partition into and permeate lipid bilayers.^3,35^ In those simulations, many lignin monomers and dimers showed accessible passive membrane transport with neutral monolignols predicted to cross membranes more readily than charged or glycosylated derivatives. The present results are also compatible with the physical feasibility of the sink-driven diffusion model in liposome and plant-cell studies.^5^ Recently, a study suggested that vesicles may participate in transporting both precursors and catalytic machinery of lignin polymerization.^6^ This requires monolignols to be enclosed by membranes at relatively high concentrations without compromising the membrane integrity. Our results show that CA is compatible with this possibility.

The results also have implications for protein-mediated pathways as typical membrane proteins are sensitive not only to specific lipid binding but also to bulk properties such as bilayer thickness, curvature stress, elasticity, and lateral pressure.^36^ Since CA produces only small changes in the bilayer structure and dynamics, it is unlikely to broadly disrupt membrane protein function triggered by large changes in the surrounding lipid matrix. However, those subtle changes may still modulate protein activities positively or negatively, which remains to be studied.

## Conclusion

In this work, we combined a series of neutron scattering techniques to provide a cross-scale view of how coniferyl alcohol interacts with model membranes. The structural measurements show that CA incorporates into the bilayer and induces a small but clearly resolved thinning effect while preserving the overall membrane architecture. NMD does not resolve CA at a unique preferred depth in the bilayer. The dynamics measurements further show that CA affects collective membrane mechanics more strongly than local lipid mobility. NSE reveals a decrease in the effective bending modulus upon CA incorporation. QENS shows that there is no systematical change in the lipid lateral diffusion coefficient, while the jump distance increases at a higher CA loading. Together, these results indicate that CA makes the membrane more compliant at collective length scales while preserving the molecular-scale lipid fluidity.

Overall, these findings show that CA can be accommodated by lipid membranes with only limited structural and dynamical changes, remaining compatible with multiple proposed monolignol transport pathways. Here, we also demonstrate that neutron scattering is a powerful tool for biological and environmental research, providing unique molecular-level insight into structure and dynamics across multiple length and time scales.^37^

## Supplementary Material



## Data Availability

The data is available upon reasonable request.

## Notes

Additional details on CA-D5 synthesis and characterization, NMD phase determination, overlaid SANS profiles, and NSE analysis (PDF)

## References

[1] Georgs V., Piili H., Gustafsson J., Xu C. (2025). A critical review on
lignin structure, chemistry, and modification towards utilisation
in additive manufacturing of lignin-based composites. Ind. Crops Prod..

[2] Miao Y. C., Liu C. J. (2010). ATP-binding cassette-like
transporters are involved
in the transport of lignin precursors across plasma and vacuolar membranes. Proc. Natl. Acad. Sci. U. S. A..

[3] Vermaas J. V., Dixon R. A., Chen F., Mansfield S. D., Boerjan W., Ralph J. (2019). Passive membrane transport
of lignin-related compounds. Proc. Natl. Acad.
Sci. U. S. A..

[4] Perkins, M. , Smith, R. A. , Samuels, L. The transport of monomers during lignification in plants: anything goes but how? Current Opinion in Biotechnology. 2019 Food Biotechnology Plant Biotechnology56:69–74. doi: 10.1016/j.copbio.2018.09.011. 30347315

[5] Perkins M. L., Schuetz M., Unda F., Chen K. T., Bally M. B., Kulkarni J. A. (2022). Monolignol export by
diffusion down a polymerization-induced
concentration gradient. Plant Cell.

[6] Kankaanpää S., Väisänen E., Goeminne G., Soliymani R., Desmet S., Samoylenko A. (2024). Extracellular vesicles
of Norway spruce contain precursors and enzymes for lignin formation
and salicylic acid. Plant Physiol..

[7] Moradipour M., Tong X., Novak B., Kamali P., Asare S. O., Lynn B. C. (2021). Interaction
of lignin dimers with model cell membranes:
A quartz crystal microbalance and molecular dynamics simulation study. Biointerphases.

[8] Qian S., Sharma V. K., Clifton L. A. (2020). Understanding
the Structure and Dynamics
of Complex Biomembrane Interactions by Neutron Scattering Techniques. Langmuir.

[9] Qian S., Zhang H., Leite W., Whitten A. E., Zolnierczuk P., Yuan Y. (2026). Perturbation of nanoplastics
on biomembranes: molecular
insights from neutron scattering. Environ. Sci:
Nano.

[10] Qian S., Rai D. K. (2018). Grazing-Angle Neutron
Diffraction Study of the Water
Distribution in Membrane Hemifusion: From the Lamellar to Rhombohedral
Phase. J. Phys. Chem. Lett..

[11] Qian S., Zolnierczuk P. A. (2022). Interaction
of a short antimicrobial peptide on charged
lipid bilayer: A case study on aurein 1.2 peptide. BBA Adv..

[12] Qian S., Nagy G., Zolnierczuk P., Mamontov E., Standaert R. (2024). Nonstereotypical
Distribution and Effect of Ergosterol in Lipid Membranes. J. Phys. Chem. Lett..

[13] Darkes M. J. M., Bradshaw J. P. (2000). Real-time swelling-series method
improves the accuracy
of lamellar neutron-diffraction data. Acta Cryst.
D.

[14] Qian S., Wang W., Yang L., Huang H. W. (2008). Structure of transmembrane
pore induced by Bax-derived peptide: Evidence for lipidic pores. Proc. Natl. Acad. Sci. U. S. A..

[15] Heller W. T., Hetrick J., Bilheux J., Calvo J. M. B., Chen W. R., DeBeer-Schmitt L. (2022). drtsans: The data reduction
toolkit for small-angle
neutron scattering at Oak Ridge National Laboratory. SoftwareX.

[16] Zaccai G., Blasie J. K., Schoenborn B. P. (1975). Neutron
Diffraction Studies on the
Location of Water in Lecithin Bilayer Model Membranes. Proc. Natl. Acad. Sci. U. S. A..

[17] Chen X., Sa’adedin F., Deme B., Rao P., Bradshaw J. (2013). Insertion
of TAT peptide and perturbation of negatively charged model phospholipid
bilayer revealed by neutron diffraction. Biochim.
Biophys. Acta.

[18] Heller W. T., Cuneo M., Debeer-Schmitt L., Do C., He L., Heroux L. (2018). The suite of small-angle
neutron scattering instruments
at Oak Ridge National Laboratory. J. Appl. Cryst,
J. Appl. Crystallogr..

[19] Qian S., Heller W. T. (2011). Peptide-Induced Asymmetric Distribution
of Charged
Lipids in a Vesicle Bilayer Revealed by Small-Angle Neutron Scattering. J. Phys. Chem. B.

[20] Ohl M., Monkenbusch M., Arend N., Kozielewski T., Vehres G., Tiemann C. (2012). The spin-echo spectrometer
at the Spallation Neutron Source (SNS). Nucl.
Instrum. Methods Phys. Res. A: Accel. Spectrom. Detect. Assoc. Equip.

[21] Zolnierczuk P. A., Holderer O., Pasini S., Kozielewski T., Stingaciu L. R., Monkenbusch M. (2019). Efficient
data extraction from neutron
time-of-flight spin-echo raw data. J. Appl.
Crystallogr..

[22] Zilman A. G., Granek R. (1996). Undulations and Dynamic Structure Factor of Membranes. Phys. Rev. Lett..

[23] Watson M. C., Brown F. L. H. (2010). Interpreting Membrane Scattering
Experiments at the
Mesoscale: The Contribution of Dissipation within the Bilayer. Biophys. J..

[24] Nagao M., Kelley E. G., Ashkar R., Bradbury R., Butler P. D. (2017). Probing
Elastic and Viscous Properties of Phospholipid Bilayers Using Neutron
Spin Echo Spectroscopy. J. Phys. Chem. Lett..

[25] Sharma V. K., Nagao M., Rai D. K., Mamontov E. (2019). Membrane softening
by nonsteroidal anti-inflammatory drugs investigated by neutron spin
echo. Phys. Chem. Chem. Phys..

[26] Mamontov E., Herwig K. W. (2011). A time-of-flight
backscattering spectrometer at the
Spallation Neutron Source, BASIS. Rev. Sci.
Instrum..

[27] Arnold O., Bilheux J. C., Borreguero J. M., Buts A., Campbell S. I., Chapon L. (2014). Mantid
Data analysis and visualization package for
neutron scattering and SR experiments. Nucl.
Instrum. Methods Phys. Res. A: Accel. Spectrom. Detect. Assoc. Equip.

[28] Azuah R. T., Kneller L. R., Qiu Y., Tregenna-Piggott P. L. W., Brown C. M., Copley J. R. D. (2009). DAVE: A Comprehensive
Software Suite for the Reduction, Visualization, and Analysis of Low
Energy Neutron Spectroscopic Data. J. Res. Natl.
Inst. Stand. Technol..

[29] Villagrana R., López-Marqués R. L. (2023). Plant transbilayer lipid asymmetry
and the role of lipid flippases. Emerg Top Life
Sci..

[30] Bryant S. J., Garvey C. J., Darwish T. A., Georgii R., Bryant G. (2024). Molecular
interactions with bilayer membrane stacks using neutron and X-ray
diffraction. Adv. Colloid Interface Sci..

[31] Glatter, O. Scattering Methods and their Application in Colloid and Interface Science, 1st ed.; Elsevier: Amsterdam, Netherlands, 2018.

[32] Lee M. T., Hung W. C., Chen F. Y., Huang H. W. (2005). Many-Body
Effect
of Antimicrobial Peptides: On the Correlation Between Lipid’s
Spontaneous Curvature and Pore Formation. Biophys.
J..

[33] Hoffmann I., Kelley E. G., Nagao M., Vlahovska P., Granek R. (2026). Describing neutron spin echo data
from undulating lipid
vesicles: recent advances. J. Appl. Crystallogr..

[34] Granek R., Hoffmann I., Kelley E. G., Nagao M., Vlahovska P. M., Zilman A. (2024). Dynamic structure factor
of undulating vesicles: finite-size
and spherical geometry effects with application to neutron spin echo
experiments. Eur. Phys. J. E.

[35] Vermaas J. V., Crowley M. F., Beckham G. T. (2022). Molecular
simulation of lignin-related
aromatic compound permeation through gram-negative bacterial outer
membranes. J. Biol. Chem..

[36] Levental I., Lyman E. (2023). Regulation of membrane protein structure
and function by their lipid
nano-environment. Nat. Rev. Mol. Cell Biol..

[37] Johs A., Qian S., Coates L., Davison B. H., Elkins J. G., Gu X. (2024). New Opportunities
for Neutrons in Environmental and
Biological Sciences. Front. Environ. Sci. Eng..

