# Supplementary material for: The Structural and Dynamical Effects of Monolignol Coniferyl Alcohol on Biomembranes
Source: ACS Omega. 2026 Oct 28;11(44):68861–9. doi: 10.1021/acsomega.6c08435 (PMC13756693; doi:10.1021/acsomega.6c08435)
Supplement: Supplementary file 1 [file ao6c08435_si_001.pdf]

*Supplemental Information for*

# The Structural and Dynamical Effects of Monolignol Coniferyl Alcohol on Biomembranes

*Shuo Qian *, Honghai Zhang, Piotr Zolnierczuk, Eugene Mamontov*

Oak Ridge National Laboratory, Oak Ridge, TN USA

* Corresponding Author

Shuo Qian, Oak Ridge National Laboratory, Oak Ridge, TN

qians@ornl.gov

865-241-1934

**Preparation procedure and product characterization for coniferyl alcohol-d5 (CA-D5):**

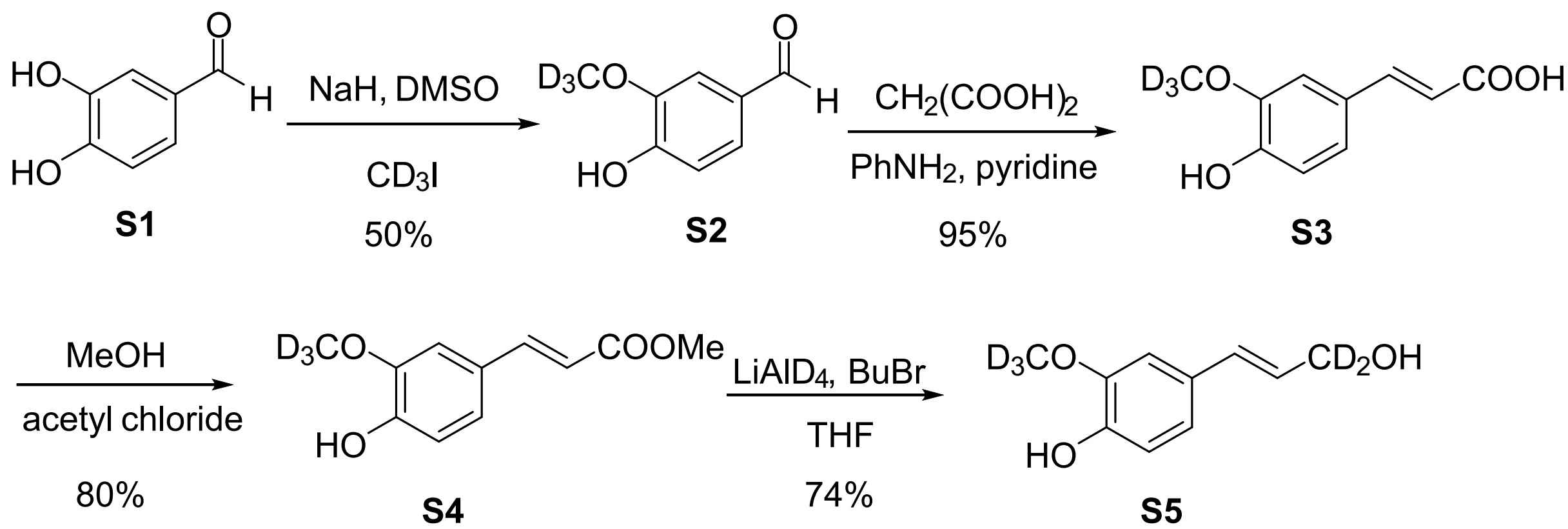


Figure S1 Synthetic route for CA-D5

*4-hydroxy-3-methoxybenzaldehyde-d3 (S2)*

In a 500 mL round bottle flask, NaH (5.28 g, 132 mmol, 60 % in mineral oil) was added slowly to a stirring solution of 3,4-dihydroxybenzaldehyde (**S1**) (8.28 g, 60 mmol) in dry DMSO (210 mL) at 0ºC. Then, the mixture was allowed to stir for 30 mins and the solution turned to clear brown color. The DMSO solution (30 mL) containing $CD_3I$ (8.76 g, 60.6 mmol) was added dropwise, and the mixture was stirred overnight. Completion of the reaction was monitored by TLC. The reaction mixture was then quenched by aq. $NH_4Cl$ solution (200 mL), washed with water (150 mL) and extracted with ether (200 mL x 3). The organic layer was dried over anhydrous sodium sulfate. The solvent was evaporated under vacuum to give a crude product, which was further purified by column chromatography on silica gel using hexane-ethyl acetate (4 : 1) mixture as eluent. Yield: 4.62 g (50 %), yellow solid, $^1H$ NMR (400 MHz, $CDCl_3$) δ 9.86 (br s, 1 H), 7.46 – 7.44 (m, 2 H), 7.07 (d, $J$ = 6.4 Hz, 1H), 6.20 (br s, 1 H).

*3-(4-hydroxy-3-methoxyphenyl)acrylic acid-d3 (S3)*

In a 100 mL round bottle flask, 4-hydroxy-3-methoxybenzaldehyde-d3 (**S2**) (3.1 g, 20 mmol), malonic acid (4.4 g, 44 mmol) and aniline (240 mg, 2.6 mmol) was dissolved in 20 mL of pyridine. The mixture was stirred at 80 °C for 24 hours. Completion of the reaction was monitored by TLC. The solution was poured into an ice cold HCl solution (4 N, 40 mL). The mixture was allowed to gently stir for 20 mins and precipitation formed. The solid was collected by filtration and used for next step without further purification. Yield: 3.74 g (95 %), yellow solid, $^1$H NMR (400 MHz, DMSO-$d_6$) δ 12.12 (br s, 1H), 9.54 (br s, 1H), 7.49 (d, $J$= 12.3 Hz, 1 H), 7.28 (s, 1 H), 7.08 (d, $J$= 5.8 Hz, 1 H), 6.78 (d, $J$= 6.4 Hz, 1H), 6.36 (d, $J$= 13.5 Hz, 1 H).

*methyl 3-(4-hydroxy-3-methoxyphenyl)acrylate-d3 (S4)*

In a 100 mL round bottle flask, acetyl chloride (1.18 g, 15.1 mmol) was added dropwise to a stirring solution of 3-(4-hydroxy-3-methoxyphenyl)acrylic acid (**S3**) (2.97 g, 15 mmol) in dry MeOH (20 mL) at 0°C. Then, the mixture was refluxed for one hour. Completion of the reaction was monitored by TLC. The organic solvent was directly evaporated under vacuum to obtain the crude product, which was further purified by column chromatography on silica gel using hexane-ethyl acetate (2 : 1) mixture as eluent. Yield: 2.54 g (80 %), yellow solid, $^1$H NMR (400 MHz, $CDCl_3$) δ 7.64 (d, $J$= 12.9 Hz, 1 H), 7.09 (d, $J$= 9.7 Hz, 1 H), 7.04 (s, 1 H), 6.94 (d, $J$= 8.1 Hz, 1H), 6.32 (d, $J$= 12.9 Hz, 1 H), 5.88 (br s, 1H), 3.82(s, 3H).

*Coniferyl alcohol-d5 (S5)*

In a 100 mL vessel, $LiAlD_4$ solution (1 M in THF, 8 mL) was added to anhydrous THF (8 mL) at 0°C followed by slow addition of *n*-bromobutane (1.1 g, 8 mmol) and stirred for 3 hours under $N_2$. Methyl 3-(4-hydroxy-3-methoxyphenyl)acrylate-d3 (**S4**) (872 mg, 4 mmol) was dissolved in THF (15 mL) and cannulated dropwise into the $LiAlD_4$ solution at -78°C. Reaction was allowed to warm slowly to room temperature and stirred overnight. Reaction is quenched by dilution with ethyl acetate, followed by the slow addition of $H_2O$ until no more gas evolves. Saturated $NH_4Cl$ is added to give a paste-like mixture.

Mixture is decanted off, followed by re-extraction of the paste with ethyl acetate. The organic phase was washed with brine, dried over sodium sulfate. The organic solvent was evaporated under vacuum to give a crude product, which was further purified by column chromatography on silica gel using hexane- ethyl acetate (2 : 1) mixture as eluent. Yield: 550 mg (74 %), yellow solid, $^1$H NMR (400 MHz, $CDCl_3$) δ 6.94 – 6.88 (m, 3 H), 6.56 (d, *J* = 14.2 Hz, 1H), 6.24 (d, *J* = 11.6 Hz, 1 H), 5.65 (br s, 1H).

All the products were characterized by nuclear magnetic resonance (NMR) instrument. NMR spectra were recorded at 23 ºC on a Varian VNMRS 400 MHz NMR spectrometer in $CDCl_3$ unless otherwise noted. Chemical shifts were determined relative to residual $CHCl_3$ (7.26 ppm) for proton. By comparing their $^1$H NMR spectrums with literature's data [1], the structures of desired products were confirmed.

**Diffraction phases determined by the swelling method**

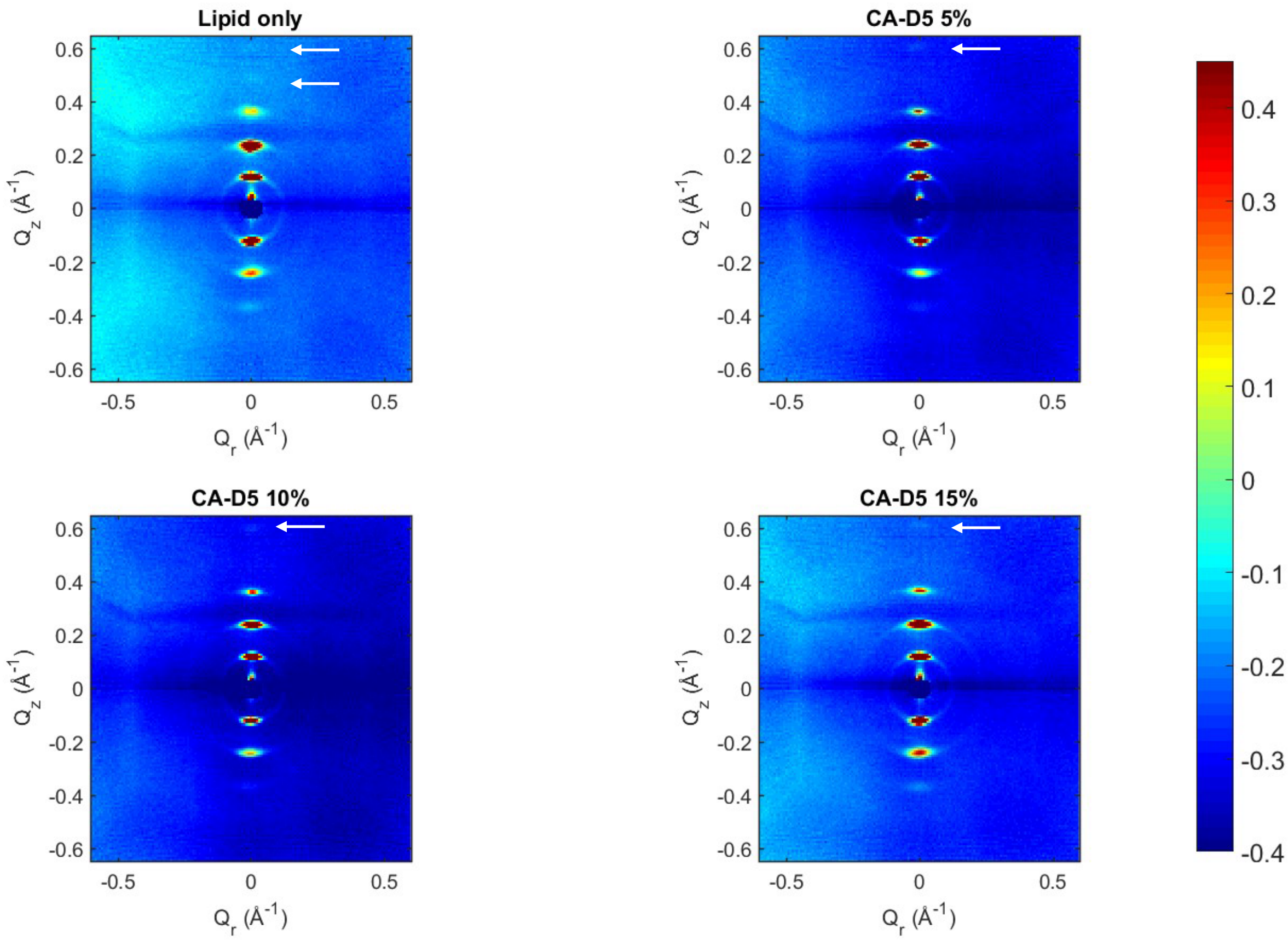


Figure S2 Diffraction patterns from the rocking scan at RH 98%. The colormap is in logarithm scale to highlight weaker peaks. The arrows show where the 4$^{th}$ or 5$^{th}$ peaks are, if they are present, which are much weaker compared to other orders.

The Lorentz factor ($C_L$) correction accounts for the reciprocal-space sampling associated with the rocking scan. The absorption ($C_{abs}$) correction accounts for attenuation through the sample. The sample geometry ($C_{geo}$) correction accounts for the changing illuminated of the sample as the substrate is rotated. [2,3]

The Lorentz factor is given by

$$C_L = 1/\sin 2\theta, \quad (2)$$

where $\theta$ is the scattering angle.

The absorption factor is given by

$C_{abs}=(1-\exp(-2\mu d/\sin\theta))/2\mu d$, (3)

where $\mu$ is the absorption coefficient of the sample, $d$ is the thickness of the sample.

$C_{geo}$ is a constant, as the sample was over-illuminated in the neutron beam and remained approximately unchanged for the angular range in the scans.

The phases were determined by the swelling method applied with data from RH 98% and 95% [4–6]. As the RH changes within a small range, the membrane structure only changes subtly with the lamellar repeat spacing and structure factor amplitudes. One can use a set of discrete structure factor amplitudes $|F(q_n)|$ from varying RHs to construct a continuous function $C(q) = \sum_1^n F(q_n) sinc(\frac{qD}{2} - \pi n)$, where D is the lamellar repeat spacing, $q_n = 2\pi n/D$. As the membrane bilayer structure is centrosymmetric, the phases for $F(q_n)$ are either +1 or -1. With the correct choice of the phases, the continuous function will show a smooth variation with the same trend of other discrete amplitudes to confirm the phases. The dataset from RH 98% was used to construct this continuous function as shown in Fig. S3. The red dataset is from RH 95%. If the trends of change between data points are consistent with the continuous curve, the choice of the phases (−1, −1, +1, −1, −1) is confirmed.

Note that we did not collect the data for 5% CA at RH 95% owing to availability of the allocated neutron beam time; however, the phases at high concentrations and lower concentrations are the same, therefore we assume the same phases can be applied for that sample.

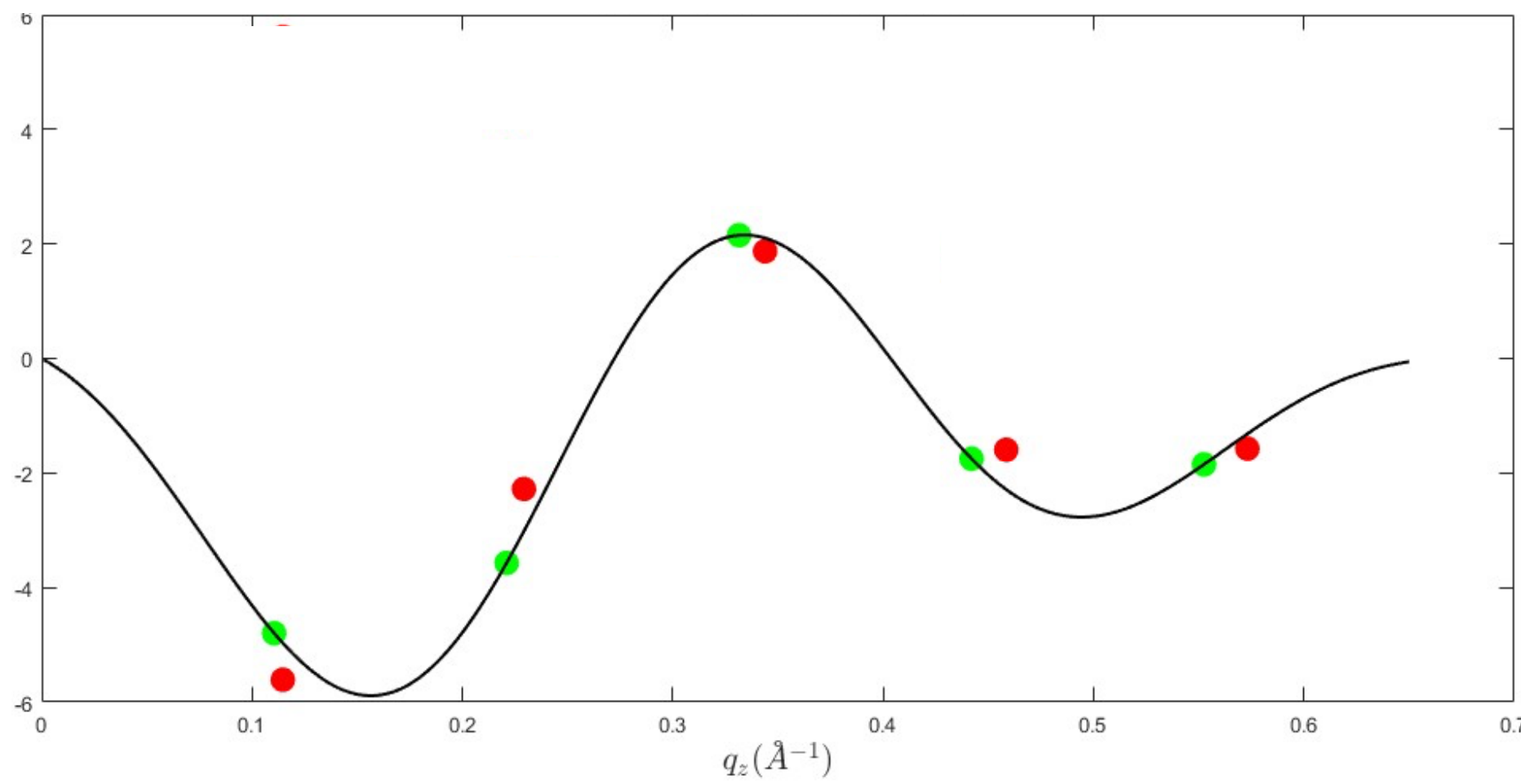


(a) Lipid only

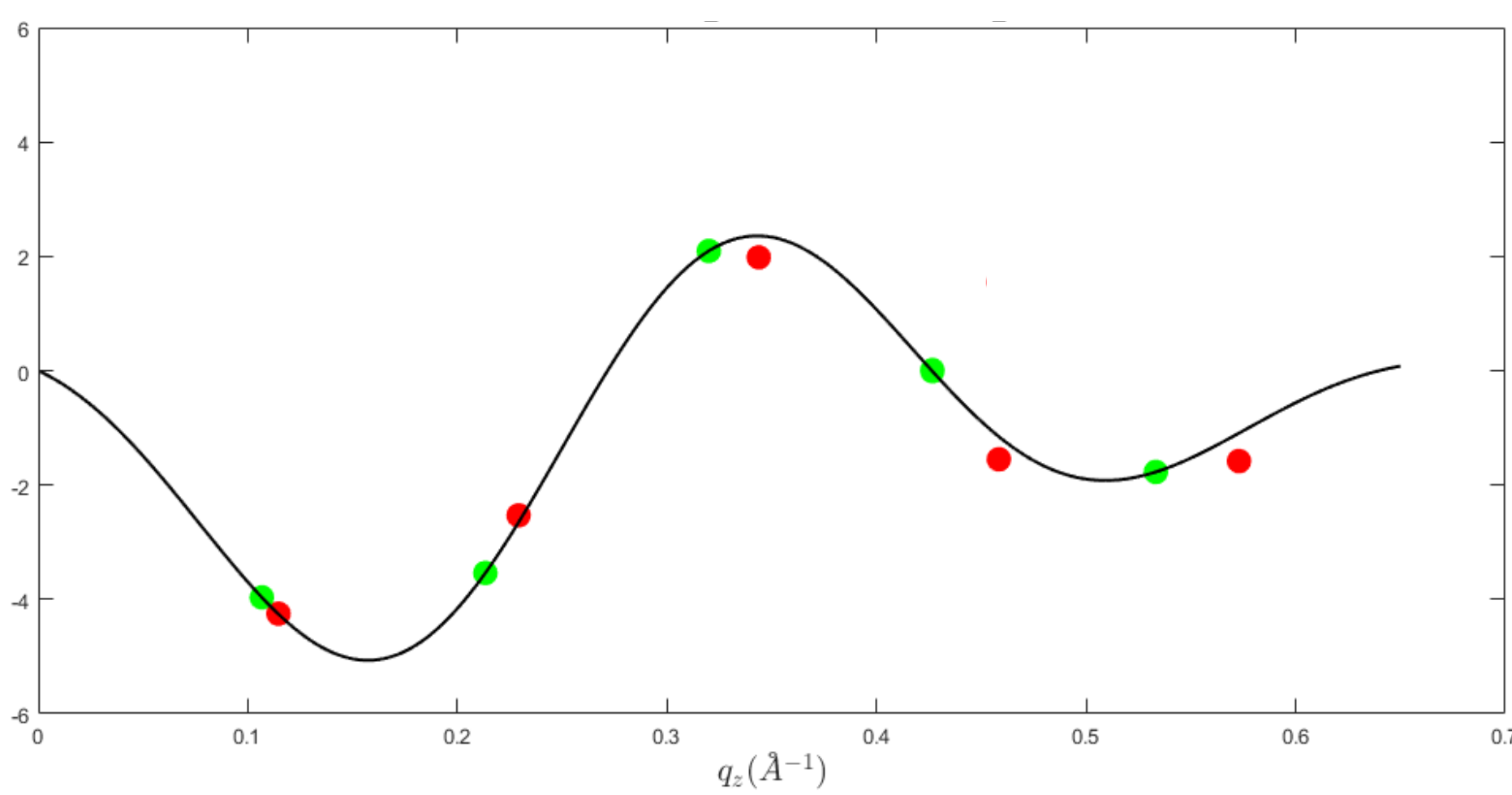


(b) 10% CA-D5 sample

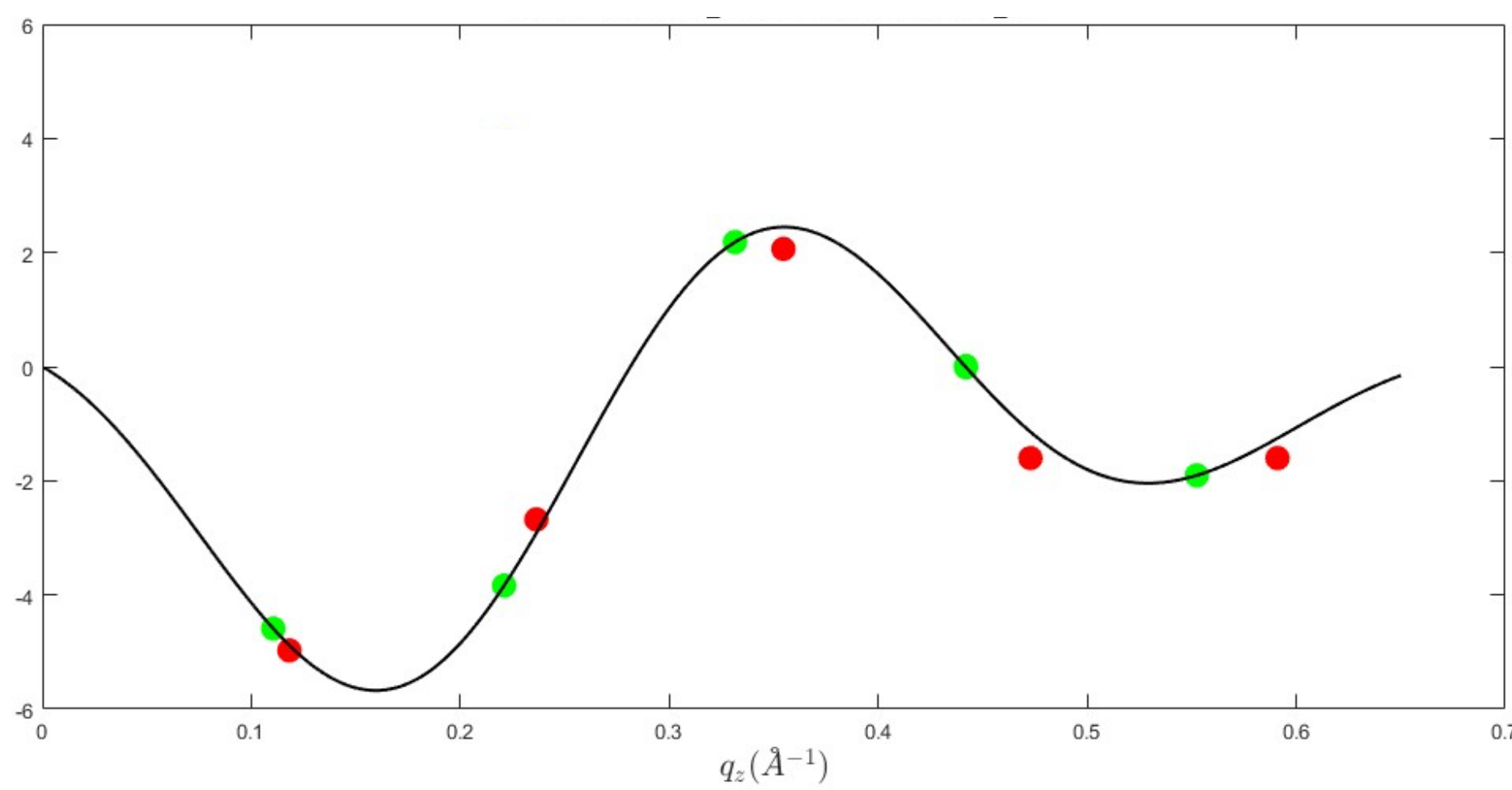


(c) 15% CA-D5 sample

Figure S3 Measured structure-factor amplitudes at 98% RH and 95% RH, plotted against Q, together with the continuous transform constructed from the 98% RH data. The solid curves are used to assign the signs of the structure factors and are not independent fits to all data points.

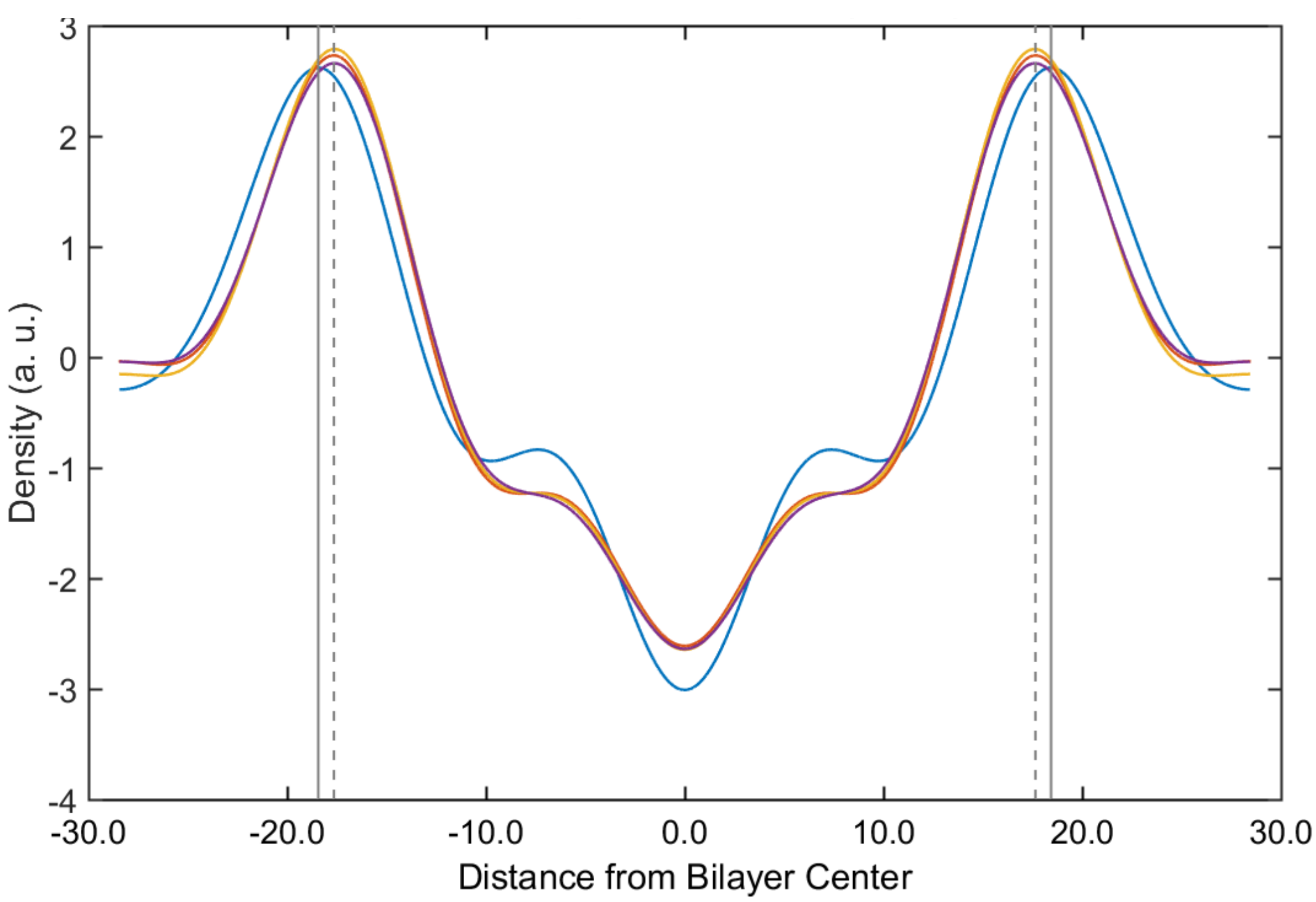


Figure S4 The overlaid SLD profiles from RH 98% (The color scheme is the same as in Fig. 2)

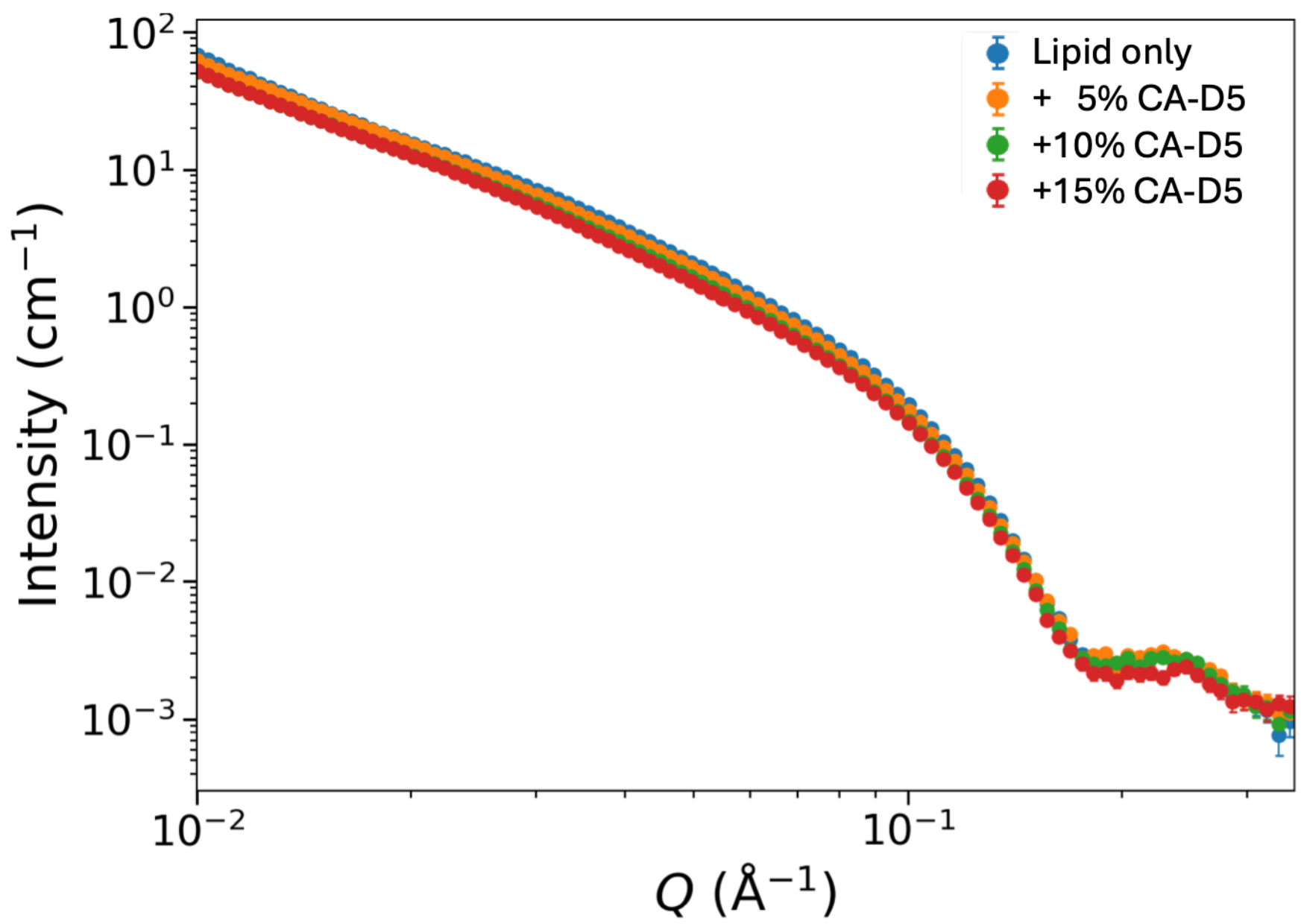


Figure S5 The overlaid SANS data

Table S1 Fitting parameters for the SANS data

| Parameter | Lipid only | +5% CA-D5 | +10% CA-D5 | +15% CA-D5 |
|---|---|---|---|---|
| **$\chi^2$/Npts** | 2.068 | 2.434 | 1.870 | 1.808 |
| **Scale** | 0.040 | 0.040 | 0.032 | 0.032 |
| **Background (cm$^{-1}$)** | 0.001 | 0.001 | 0.0011 | 0.0008 |
| **Core SLD ($10^{-6}$ Å$^{-2}$)** | 6.34 fixed | 6.34 fixed | 6.34 fixed | 6.34 fixed |
| **Core radius (Å)** | 500 fixed | 500 fixed | 500 fixed | 500 fixed |
| **Solvent SLD ($10^{-6}$ Å$^{-2}$)** | 6.34 fixed | 6.34 fixed | 6.34 fixed | 6.34 fixed |
| **Number of shells, n** | 3 fixed | 3 fixed | 3 fixed | 3 fixed |
| **Shell 1 SLD ($10^{-6}$ Å$^{-2}$)** | 2.0 fixed | 2.0 fixed | 2.0 fixed | 2.0 fixed |
| **Shell 1 thickness (Å)** | 9.40 ± 0.04 | 9.66 ± 0.04 | 9.70 ± 0.05 | 9.10 ± 0.04 |
| **Shell 2 SLD ($10^{-6}$ Å$^{-2}$)** | −0.4 fixed | −0.4 fixed | −0.4 fixed | −0.4 fixed |
| **Shell 2 thickness (Å)** | 26.05 ± 0.05 | 24.36 ± 0.05 | 24.40 ± 0.05 | 24.20 ± 0.05 |
| **Shell 3 SLD ($10^{-6}$ Å$^{-2}$)** | 2.0 fixed | 2.0 fixed | 2.0 fixed | 2.0 fixed |
| **Shell 3 thickness (Å)** | 5.00 ± 0.04 | 5.10 ± 0.04 | 5.00 ± 0.06 | 5.05 ± 0.04 |
| **Radius polydispersity** | 0.25 fixed | 0.25 fixed | 0.25 fixed | 0.25 fixed |
| Radius distribution | Schulz | Schulz | Schulz | Schulz |

**Neutron Spin Echo**

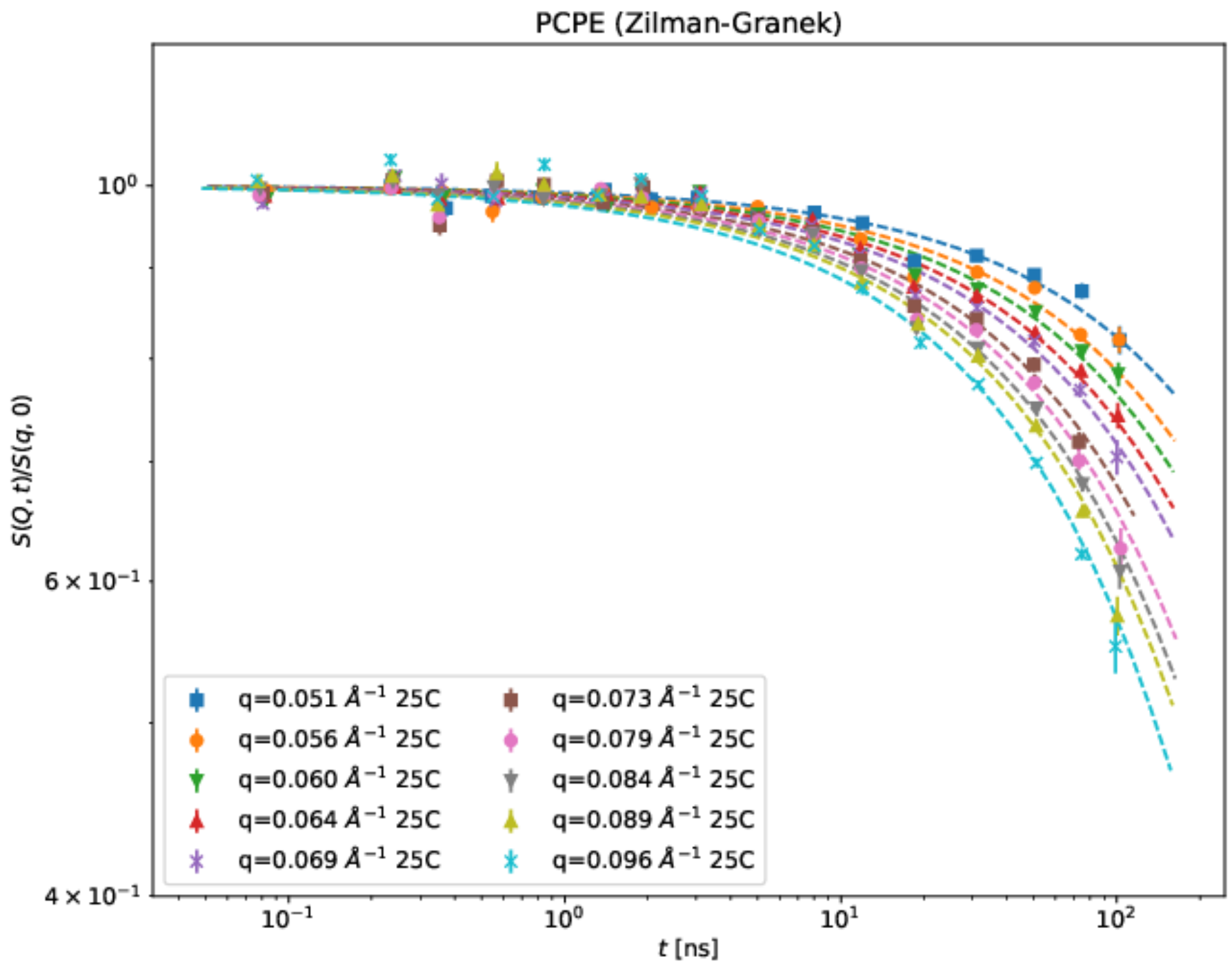


(a) Lipid only

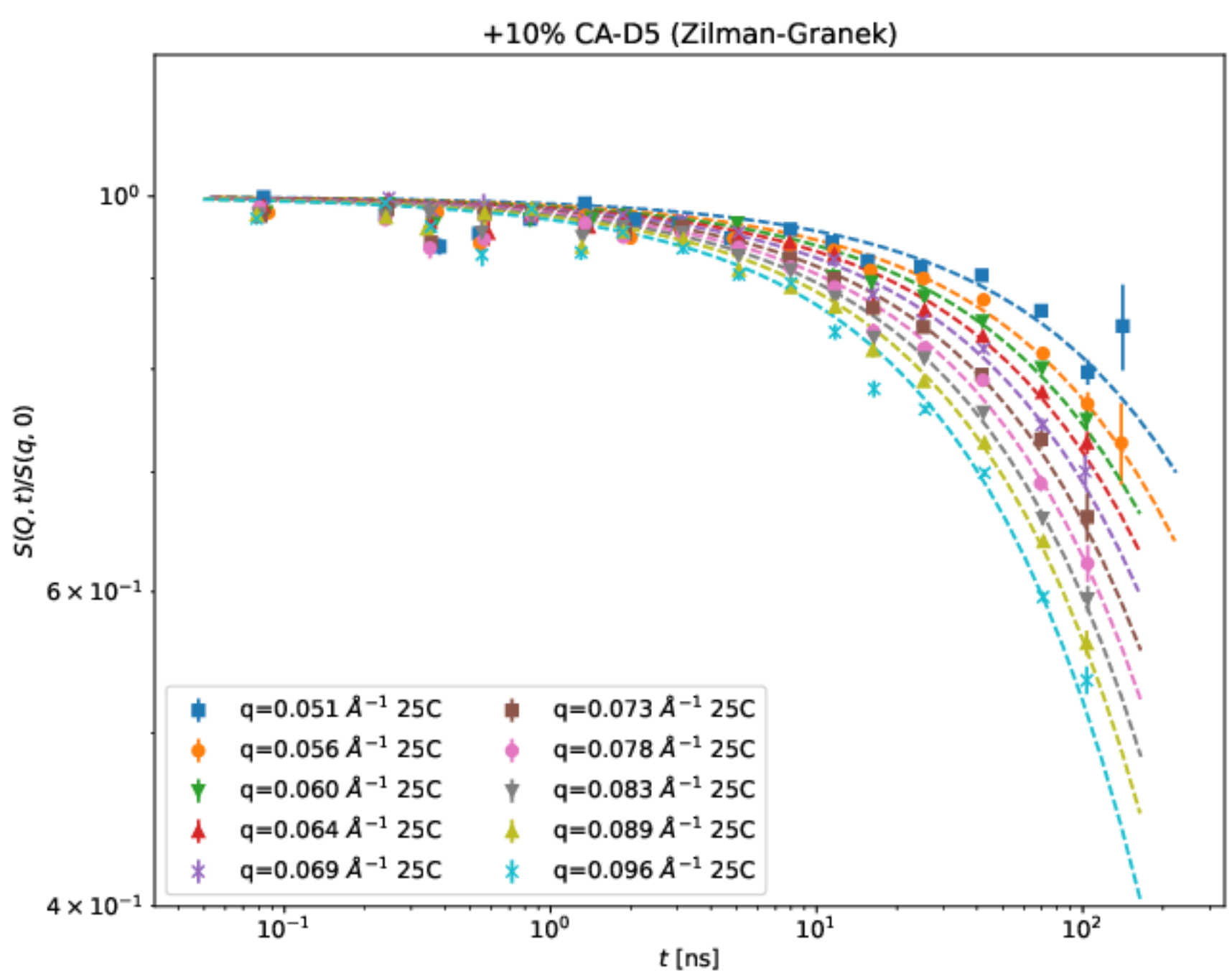


(b) 10% CA-D5

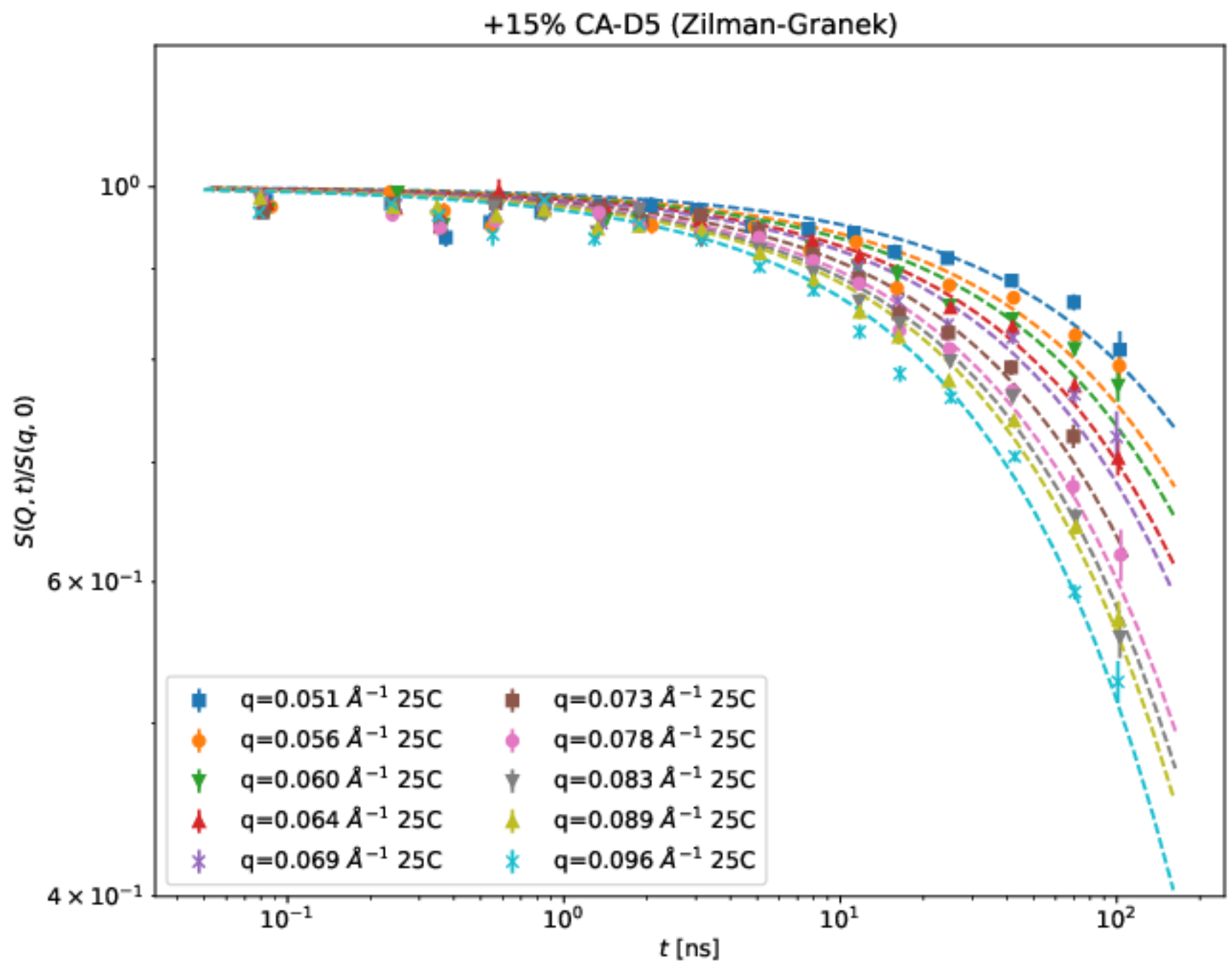


(c) 15% CA-D5

Figure S6 S(Q,t)/S(Q,0) Intermediate scattering function for the measured samples.

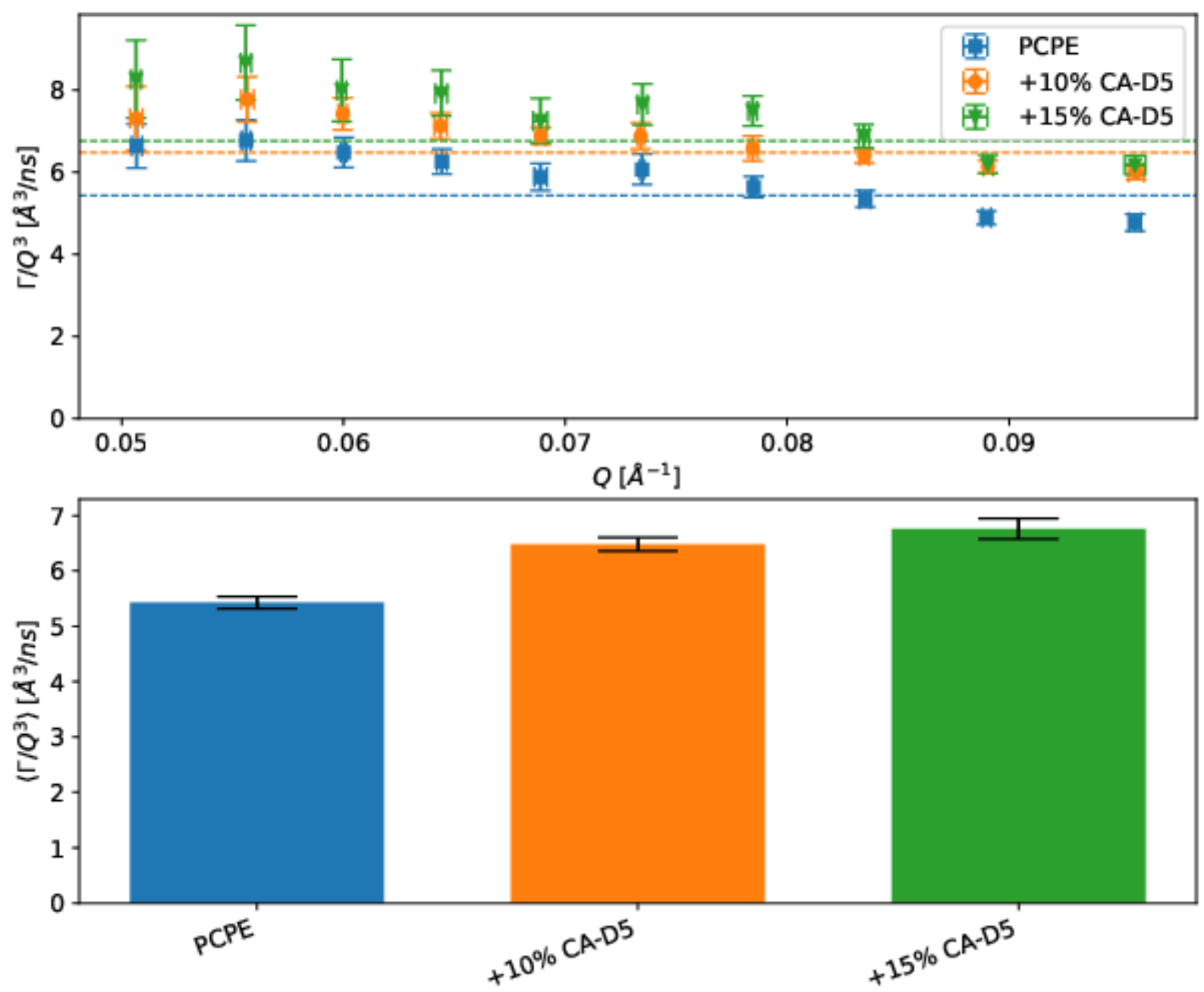


Figure S7 Top: Membrane Q-dependent relaxation rates Γ(Q) normalized to $Q^3$ as a function of Q; Bottom: their average values of $\Gamma(Q)/Q^3$ over the Q range presented in the top panel

Once the effective bending modulus, $\tilde{\kappa}$, is determined.

Following Watson and Brown [7] and Nagao *et al*. [8], the effective bending modulus, $\tilde{\kappa}$, and the intrinsic bending modulus, $\kappa$, are related by

$$\kappa = \frac{\tilde{\kappa}}{1 + 48\left(\frac{h}{2h_c}\right)^2} = \frac{\tilde{\kappa}}{13}$$

where $h$ and $h_c$ are the distances from the bilayer midplane to the neutral surface and the monolayer hydrocarbon thickness, respectively. The ratio $h/h_c$ is commonly assumed to be $1/2$.

Finally, the bilayer area compressibility modulus, $K_A$, is obtained from

$$K_A = \frac{\beta\kappa}{(2h_c)^2},$$

where the so-called coupling constant, $\beta = 24$, is taken from the literature [9]. $2h_C$ are from the SANS chain thickness results.

Table S2 Relaxation rates $\Gamma(Q)/Q^3$, bending modulus $\tilde{\kappa}$, and compressibility moduli $K_A$ for the measured samples.

| Sample | $2h_C$ [Å] | T [°C] | $\eta_{D2O}$ [cP] | $\Gamma(Q)/Q^3$ [Å$^3$/ns] | $\tilde{\kappa}$ [kT] | $K_A$ [mN/m] |
|---|---|---|---|---|---|---|
| PCPE lipid only | 26.05 ± 0.05 | 25.0 ± 0.5 | 1.10 | 5.43 ± 0.11 | 297 ± 12 | 332 ± 15 |
| +10% CA-D5 | 24.40 ± 0.05 | 25.0 ± 0.5 | 1.10 | 6.48 ± 0.12 | 209 ± 8 | 266 ± 11 |
| +15% CA-D5 | 24.20 ± 0.05 | 25.0 ± 0.5 | 1.10 | 6.76 ± 0.19 | 192 ± 11 | 249 ± 15 |

**References:**


(1) Lairez, D.; Cathala, B.; Monties, B.; Bedos-Belval, F.; Duran, H.; Gorrichon, L. Aggregation during Coniferyl Alcohol Polymerization in Pectin Solution:  A Biomimetic Approach of the First Steps of Lignification. *Biomacromolecules* **2005**, *6* (2), 763–774. https://doi.org/10.1021/bm049390y

(2) Qian, S.; Rai, D. K. Grazing-Angle Neutron Diffraction Study of the Water Distribution in Membrane Hemifusion: From the Lamellar to Rhombohedral Phase. *J. Phys. Chem. Lett.* **2018**, *9* (19), 5778–5784. https://doi.org/10.1021/acs.jpclett.8b01602

(3) Yang, L.; Harroun, T. A.; Heller, W. T.; Weiss, T. M.; Huang, H. W. Neutron Off-Plane Scattering of Aligned Membranes. I. Method of Measurement. *Biophysical Journal* **1998**, *75* (2), 641–645. https://doi.org/10.1016/S0006-3495(98)77554-X
(4) Torbet, J.; Wilkins, M. H. X-Ray Diffraction Studies of Lecithin Bilayers. *J Theor Biol* **1976**, *62* (2), 447–458. https://doi.org/10.1016/0022-5193(76)90129-6
(5) Blaurock, A. E. Structure of the Nerve Myelin Membrane: Proof of the Low-Resolution Profile. *J. Mol. Biol.* **1971**, *56* (1), 35–52. https://doi.org/10.1016/0022-2836(71)90082-9
(6) Darkes, M. J. M.; Bradshaw, J. P. Real-Time Swelling-Series Method Improves the Accuracy of Lamellar Neutron-Diffraction Data. *Acta Cryst D* **2000**, *56* (1), 48–54. https://doi.org/10.1107/S090744499901375X
(7) Watson, M. C.; Brown, F. L. H. Interpreting Membrane Scattering Experiments at the Mesoscale: The Contribution of Dissipation within the Bilayer. *Biophysical Journal* **2010**, *98* (6), L9–L11. https://doi.org/10.1016/j.bpj.2009.11.026
(8) Nagao, M.; Kelley, E. G.; Ashkar, R.; Bradbury, R.; Butler, P. D. Probing Elastic and Viscous Properties of Phospholipid Bilayers Using Neutron Spin Echo Spectroscopy. *J. Phys. Chem. Lett.* **2017**, *8* (19), 4679–4684. https://doi.org/10.1021/acs.jpclett.7b01830
(9) Rawicz, W.; Olbrich, K. C.; McIntosh, T.; Needham, D.; Evans, E. Effect of Chain Length and Unsaturation on Elasticity of Lipid Bilayers. *Biophysical Journal* **2000**, *79* (1), 328–339. https://doi.org/10.1016/S0006-3495(00)76295-3
